# Metabolomic and transcriptomic analyses reveal the regulation of pigmentation in the purple variety of *Dendrobium officinale*

**DOI:** 10.1038/s41598-020-74789-0

**Published:** 2020-10-19

**Authors:** Xinqiao Zhan, Jufeng Qi, Bin Zhou, Bizeng Mao

**Affiliations:** 1 Institute of Biotechnology, Zhejiang University, Hangzhou, 310058 China; 2 Institute of Biopharmaceuticals, Taizhou University, Taizhou, 318000 China; 3 Zhejiang Baihua Landscape Group Co., Ltd., Taizhou, 318000 China

**Keywords:** Secondary metabolism, Plant sciences, Plant physiology

## Abstract

We performed an integrated analysis of the transcriptome and metabolome from purple (Pr) and normal cultivated varieties (CK) of *Dendrobium officinale* to gain insights into the regulatory networks associated with phenylpropanoid metabolism and to identify the key regulatory genes of pigmentation*.* Metabolite and transcript profiling were conducted by ultra-performance liquid chromatography electrospray tandem mass spectrometry (UPLC-ESI-MS/MS) and RNA sequencing. Pr had more flavonoids in the stem than did CK. Metabolome analyses showed that 148 differential metabolites are involved in the biosynthesis of phenylpropanoids, amino acids, purines, and organic acids. Among them, the delphinidin and quercetin derivatives were significantly higher in Pr. A total of 4927 differentially expressed genes (DEGs) were significantly enriched (*p* ≤ 0.01) in 50 Gene Ontology (GO) terms. Kyoto Encyclopedia of Genes and Genomes (KEGG) enrichment analyses revealed significantly enriched phenylpropanoid biosynthesis and phytohormone signal transduction in Pr versus CK. The expression levels of *flavanone 3-hydroxylase* (*F3H*) and *leucoanthocyanidin dioxygenase* (*LDOX*) affected the flux of dihydroflavonol, which led to a color change in Pr. Moreover, DEG enrichment and metabolite analyses reflected flavonoid accumulation in Pr related to brassinosteroid (BR) and auxin metabolism. The results of this study elucidate phenylpropanoid biosynthesis in *D. officinale*.

## Introduction

*Dendrobium* is the second largest genus in Orchidaceae, and contain more than 1500 species of high ornamental and medicinal value, distributed across Asia and Oceania. *Dendrobium officinale* is a popular health product in China because its active components and health-promoting effects^1,2^. Most of the active medicinal components of *Dendrobium* are polysaccharides^3,4^, with immunomodulatory and hepatoprotective activities^5^. *Dendrobium* also contains complex secondary metabolites, including alkaloids, phenols, terpenes, coumarins, and flavonoids^6,7^. In recent years, transcriptome and metabolomics technologies have been used to investigate the biosynthesis of secondary metabolites in several *Dendrobium* species. Eight flavonoid glycosides were identified by ultra-high performance liquid-chromatography–mass spectrometry in *D. catenatum* from three different locations (Zhejiang, Guangxi and Guangdong) in China. Vicenin, 2″-*O*-glucopyranosylvitexin, and schafoside were present in all the *D. catenatum* samples. Violanthin and 6,8-di-C-α-l-arabinosylapigenin were only present in Guangdong samples, while the vicenin II content was remarkably high in the Zhejiang samples^8^. The polysaccharide and alkaloid contents of *D. huoshanense* are associated with mevalonate (MVA) and 2-C-methyl-D-erythritol 4-phosphate (MEP) pathways^6^. Moreover, studies of stress resistance in *Dendrobium* have shown that the major CIPK gene transcript is affected by the biological clock under abiotic stress and is also associated with signal transduction and energy metabolism^9^. However, no studies have investigated the regulation of pigmentation in *Dendrobium*.

Flavonoids are a class of widely distributed secondary metabolites that play an important role in biotic and abiotic stresses^10,11^. Flavonoids are synthesized through phenylpropanoid metabolism, transforming phenylalanine into naringenin, which then enters the anthocyanin, flavone, and flavonol biosynthesis pathways^12^. Anthocyanins and their derivatives have been identified in numerous flowers, fruits, leaves, stems, and seeds. More than 600 naturally occurring anthocyanins are derived from three major anthocyanins: pelargonidin, cyanidin, and delphinidin. These pigments contribute to the colors orange to red, red to magenta, and magenta to purple, respectively^13^. Anthocyanin biosynthesis is a branch of general flavonoid metabolism for which biosynthetic enzymes were identified that has been reviewed extensively. Two dihydroflavonols, dihydrokaempferol and dihydroquercetin, serve as major precursors that can be catalyzed into leucoanthocyanidins by dihydroflavonol 4-reductase (DFR). Subsequently, anthocyanidin synthase (ANS) is responsible for the formation of anthocyanidins from the colorless leucoanthocyanidins^14^. Although the biosynthetic pathway of anthocyanins has been identified, the precise relationship between anthocyanin metabolism and the regulation of color change remains unresolved. This is largely because of the diversity of anthocyanin species and differences between plant varieties.

Flavonoid biosynthesis is influenced by external factors such as UV-B light, pathogens, temperature, wounds, and phytohormones^15–17^. Flavonoids are synthesized in the cytosol and transported to the vacuole for storage^18^. The vacuolar sequestration of flavonoids involves vesicle trafficking, membrane transporters, and glutathione S-transferase (GST)^19^. There is an increasing body of evidence indicating that flavonoids accumulation are tightly related with phytohormone signals in plant growth. Certain flavonoids, such as kaempferol, quercetin, and apigenin, have been shown to inhibit auxin transport and improve localized auxin level in plants^20^. In the *transparent testa4* (*tt4*) mutant, auxin transport from the shoot to the root was found to be enhanced compared to that in the wild-type^21^. Ethylene has a repressed function similar to that of auxin and modulates the flavonoids in response to gravity^22^. In addition, ethylene-induced flavonol accumulation in guard cells moderates ABA-mediated stomatal closure^23^. ABA signaling regulates the flavonoid biosynthesis by transcription factors HY5 and MYB^17^. *MYB12* and *MYB111* can be activated by HY5, and then mediate flavonol synthase expression^17^. ABA accumulation during fruit ripening stimulates the MYB-bHLH-WD40 complex transcript levels to upregulate the expression of anthocyanin biosynthesis genes^24^. Recent researches reveal that flavonoid may regulate the ABA-signaling network by detoxifying reactive oxygen species and enhancing ABA biosynthesis^25^. The flavonoid pathway transcription factor TT8 directly regulates jasmonic acid (JA) and brassinosteroid (BR) biosynthesis^26^. In Arabidopsis, JAZ interact with the MYB-bHLH-WD40 complex to regulate JA-mediated anthocyanin biosynthesis^27^. BR enhanced expression1 (BEE1) factor is an early response BR signaling component required for the full BR response. BEE1 is also involved in cold stress responses by regulating anthocyanin accumulation^28^. Furthermore, BEE1 is regulated by other phytohormones, such as ABA, which represses the transcription of BEE1, and auxins and ethylene, which enhance BE1 expression^29^. Therefore, the crosstalk between flavonoids and phytohormone metabolism play an important role in plant development.

The combination of genetic studies and multiple-omics data represents an efficient approach to decipher the regulatory networks of the active medicinal components in *Dendrobium*. However, this method is hampered by the lack of cultivars with different phenotype traits*.* In this study, metabolomic and transcriptome analyses were combined to investigate changes in the phenylpropanoid metabolism (and the related genes) of a novel purple variety of *D. officinale*. We explain the complex metabolic networks underlying biochemical traits in the purple variety (i.e., pigmentation) and provide new insights into the development of active medicinal components in *Dendrobium.*

## Results

### Pigment accumulation in the *D. officinale* purple variety (Pr) stem

**Figure 1:**
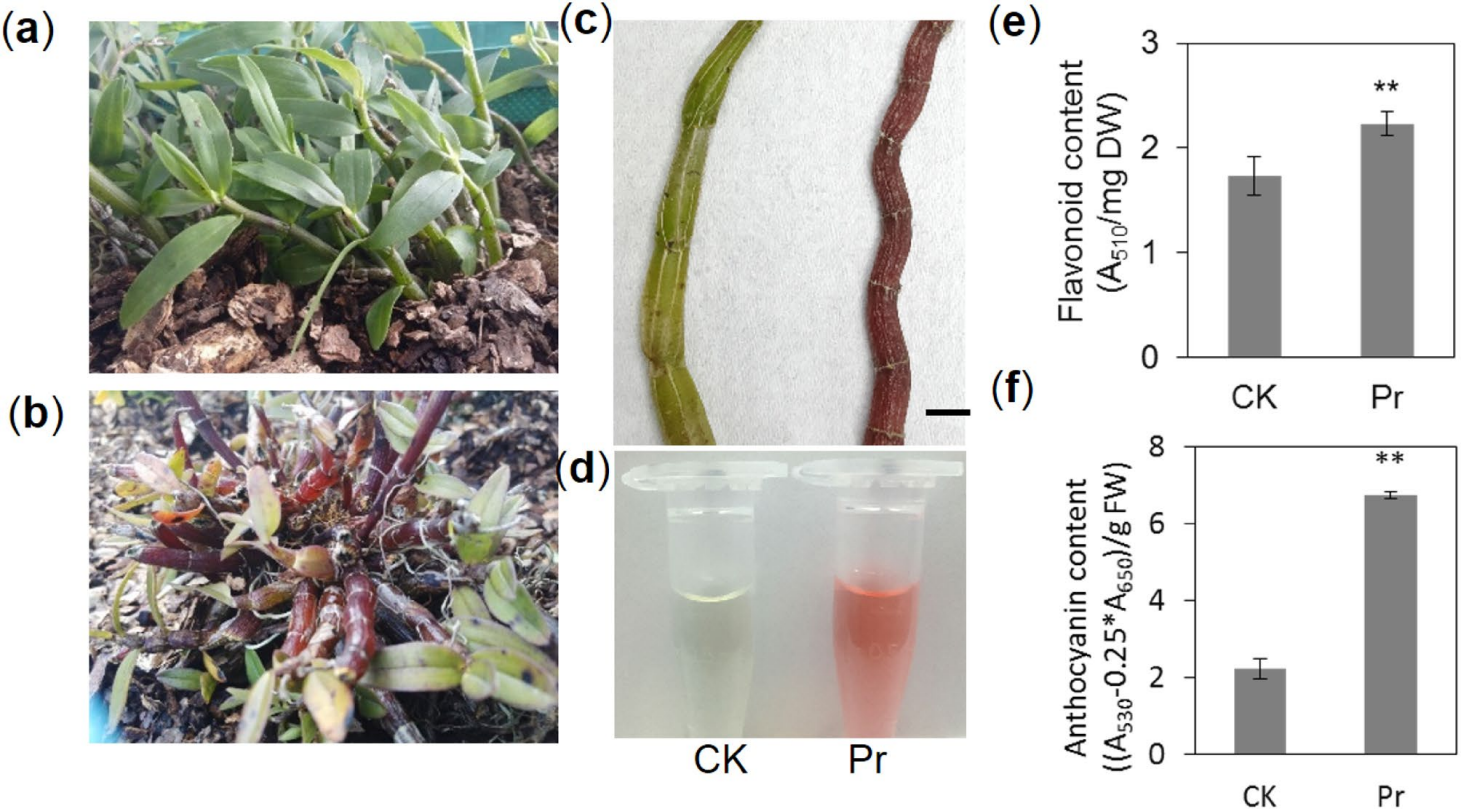
Pigment accumulation in the *D. officinale* purple variety (Pr) stem*.* (**a**) Normal varieties (CK) of *D. officinale.* (**b**) Purple varieties (Pr) of *D. officinale.* (**c**) Stem of Nr and Pr. (**d**) Methanol extracts of CK and Pr. (**e**) Flavonoid content of CK and Pr. (**f**) Anthocyanin content of CK and Pr. Values are mean ± SD (*n* = 6 independent measurements), **p < 0.01, Student's *t*-test.

Pigment variation in the purple variety (Pr) stem differed from that in the normal cultivated variety (CK) stem (Fig. 1a–c). Red pigments were observed in the Pr methanol HCl (hydrochloric acid in methanol) extracts of the stem (Fig. 1d). We also measured the pigment content of methanol extracts and found that the flavonoid and anthocyanin contents were 28.8% and 203.1% higher, respectively, in Pr than in CK (Fig. 1e,f). The extracts were analyzed by thin-layer chromatography (TLC) and exhibited chromatographic bands corresponding to the flavonoids rutin, quercetin, and myricetin (Figure S1). These results indicate that Pr accumulated more flavonoids in the stem than did CK.

### Nontargeted metabolomic analysis and overall metabolite identification

**Figure 2:**
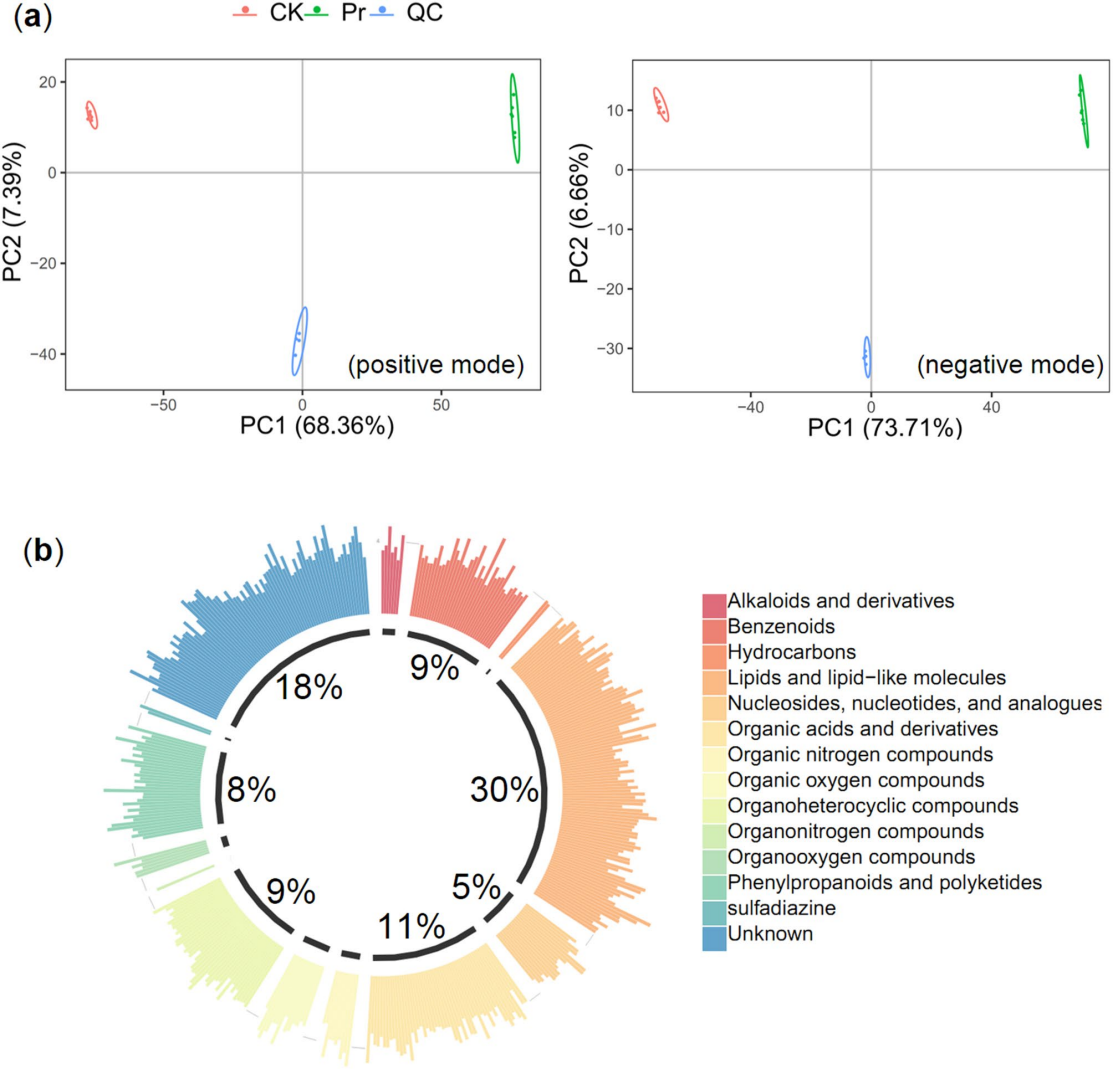
Overall analysis of metabolomics data. (**a**) PCA analysis of the two samples (Pr, green; CK, red) and quality control samples (QC, blue). The x-axis represents the first principal component and the y-axis represents the second principal. (**b**) Component analysis of the identified metabolites.

To further explore differences in the metabolite content of CK and Pr, the total extracts were subjected to ultrahigh-pressure liquid chromatography triple quadrupole mass spectrometry (UPLC-TQMS) for nontargeted metabolomics. The repeatability of CK and Pr extracts was judged by an overlapping analysis of the total ion current (TIC) in the quality control (QC) samples in positive and negative modes (Figure S2). A principal component analysis (PCA) was used to analyze the contribution rate of the first two components, which were 75.75% and 80.37% in positive and negative modes, respectively. The three period materials were clearly separated, and each formed a cluster in positive and negative modes (Fig. 2a).

After quality validation, 464 metabolites were identified in CK and Pr from the MS^2^ spectral data (Table S2). Superclass information from the Kyoto Encyclopedia of Genes and Genomes (KEGG) database was used to classify the metabolites, and the first 20 pathways are presented in Figure S3. Most of the metabolite classifications were connected to phenylpropanoid metabolism, and the largest proportions were benzenoids (9%), lipids (30%), nucleotides (5%), organic acids (11%), organoheterocyclic compounds (9%), and phenylpropanoids (8%) (Fig. 2b).

### Identification of the differentially accumulated metabolites

**Figure 3:**
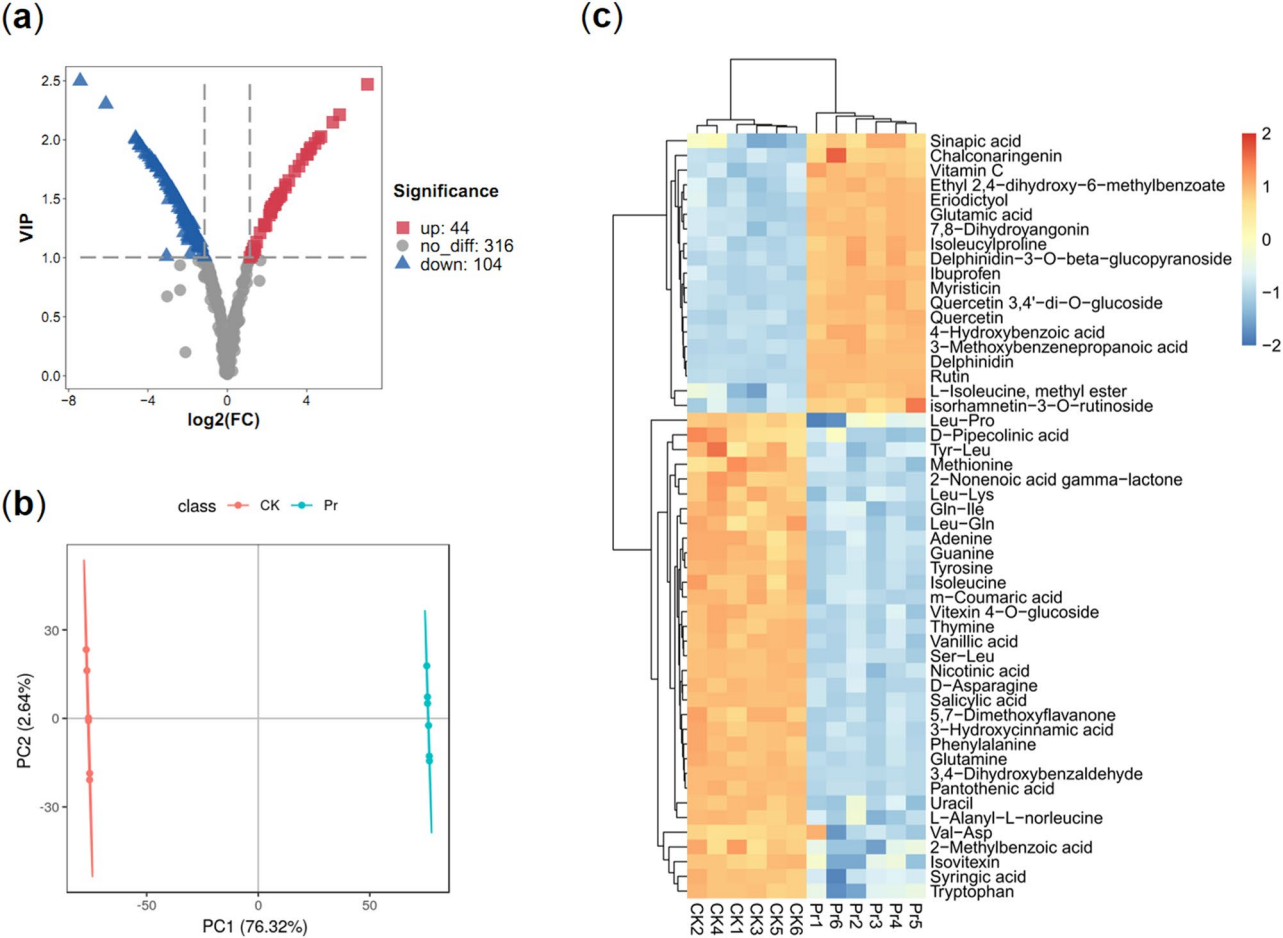
Differentially accumulated metabolites (DAMs) between Pr and CK. (**a**) Volcano plot showing the differential metabolites in Pr vs. CK. (**b**) Partial least-squares discriminant analysis (PLS-DA) of Pr and CK. (**c**) Heat map representing the hierarchical cluster analysis (HCA) in Pr vs. CK.

Differentially accumulated metabolites (DAMs) were defined by a fold change ≥ 2 or ≤ 0.5, and a variable importance in project (VIP) ≥ 1 between Pr and CK (*p* < 0.05). A total of 148 DAMs were identified (Table S3), and volcano plots were generated to show that 44 of 148 (29.7%) were up-regulated and 104 of 148 (70.3%) were down-regulated (Fig. 3a). A hierarchical cluster analysis (HCA) was also performed to assess the DAMs and QC sample accumulation patterns (Figure S4). Partial least-squares discriminant analysis (PLS-DA) was used to observe differences in the metabolic compositions of the two materials, and each formed a cluster (Fig. 3b). Based on the KEGG classifications, we created a HCA heat map to investigate the metabolites involved in phenylpropanoid, amino acid, purine, and organic acid metabolism in Pr and CK. The majority of the flavonoids were accumulated at significantly higher levels in Pr, while most of the amino acids, organic acids, and purines accumulated at significantly lower levels in Pr (Fig. 3c).

### Transcriptome data analysis

To analyze the gene expression patterns of the *D. officinale* purple variety, the raw reads from two transcript libraries were qualified, and the adapters were removed, yielding 38.48 GB of sequence data (21.03 GB from CK and 17.45 GB from Pr). The GC percentage ranged from 46 to 47%. The percentage of clean reads ranged from 88.89 to 95.40%. The probability of incorrect base calling was used to evaluate the sequencing quality according to the value of Q30. High Q30 proportions (97%) indicated high-quality RNA sequence (RNA-seq) data (Table S1). PCA revealed that Pr and CK presented different gene expression patterns. Pr clustered away from CK in PCA1, which explains 64.23% of the variation (Figure S5a). Moreover, the HAC showed that the Pr and CK gene expression profiles of the three independent biological replicates clustered together (Figure S5b).

### Identification and functional enrichment analysis of the DEGs

**Figure 4:**
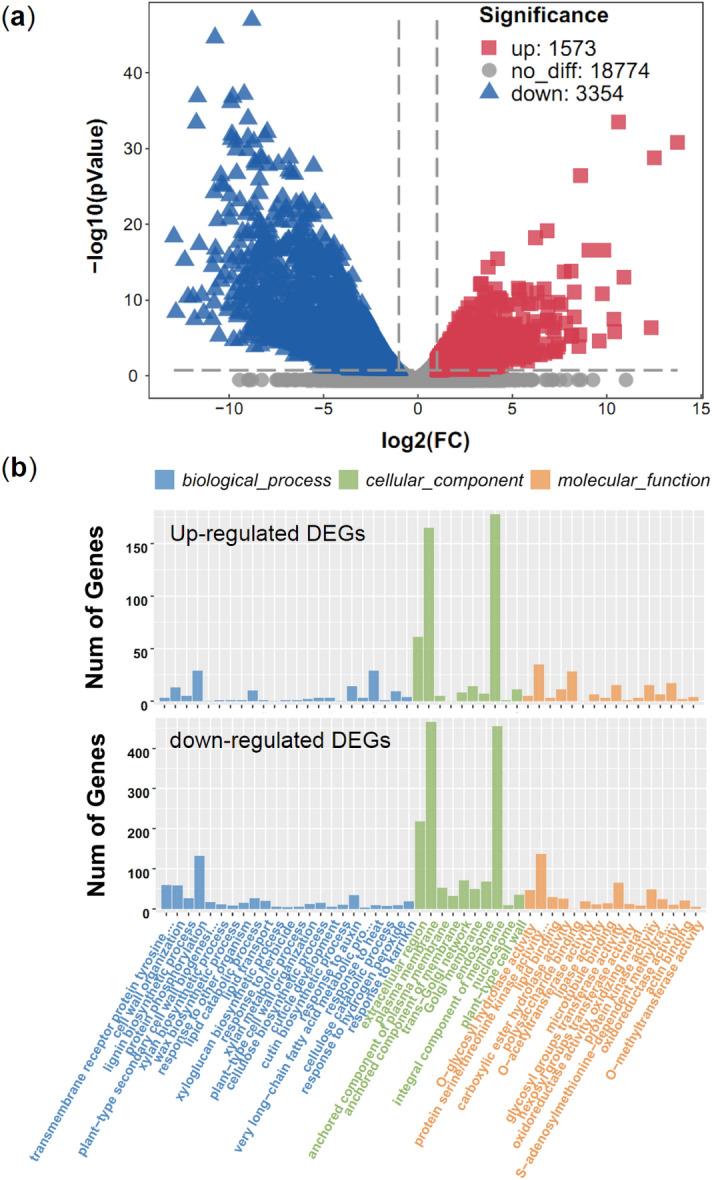
Identification of the DEGs between Pr and CK. (**a**) Volcano plot showing the differential genes in Pr vs. CK. (**b**) The Go classification of the DEGs in Pr vs. CK.

The transcript abundances of each gene from the CK and Pr data were analyzed using the fragments per kilobase of transcript per million mapped reads (FPKM) method. A false discovery rate (FDR) ≤ 0.05 and fold change (FC) > 1 were used as cutoffs to identify differentially expressed genes (DEGs). In total, 1573 genes were up-regulated and 3354 genes were down-regulated in Pr vs. CK, respectively (Fig. 4a).

In our study, the DEGs were evaluated using GO and KEGG pathway analyses. The number of DGEs in Pr vs. CK are summarized in three main GO categories (Fig. 4b, Table S4). In detail, 18 GO terms were significantly enriched (*p* < 0.005) in the biological process category in Pr vs. CK. Three of these GO terms had the largest DEGs, including “transmembrane receptor protein tyrosine kinase signaling pathway” (3 up-regulated and 59 down-regulated), “cell wall organization” (13 up-regulated and 58 down-regulated), and “protein phosphorylation” (29 up-regulated and 132 down-regulated). In the cellular components category, eight GO terms were significantly enriched (*p* < 0.005) in the Pr vs. CK comparison. In particular, 631 DEGs were enriched in the “plasma membrane” GO term, with 165 up-regulated and 466 down-regulated. A total of 633 DEGs were enriched in the “integral component of membrane” GO term, with 178 up-regulated and 455 down-regulated genes. In the molecular functions category, 10 GO terms were significantly enriched (*p* < 0.005) in Pr vs. CK. Among them, “protein serine/threonine kinase activity” and “glycosyl groups transferase activity” respectively enriched 172 DEGs (35 up-regulated and 137 down-regulated) and 80 DEGs (15 up-regulated and 65 down-regulated). GO terms associated with lipid and wax metabolism were also observed in the DEGs (Figure S6, Table S4). Four GO terms were significantly enriched (*p* < 0.005) in Pr vs. CK, including “lipid catabolic process” (*p* = 8.794E−04), “wax biosynthetic process” (*p* = 1.874E−04), “lipid transport” (*p* = 1.210E−03), and “lipid binding” (*p* = 1.716E−05). Five GO terms were significantly enriched (*p* < 0.05) in Pr vs. CK, including “very long-chain fatty acid metabolic process” (*p* = 5.470E−03), “galactolipid metabolic process” (*p* = 1.867E−02), “fatty acid biosynthetic process” (*p* = 4.087E−02), “very long-chain fatty acid biosynthetic process” (*p* = 4.440E−02), and “long-chain fatty acid-CoA ligase activity” (*p* = 4.440E−02).

**Figure 5:**
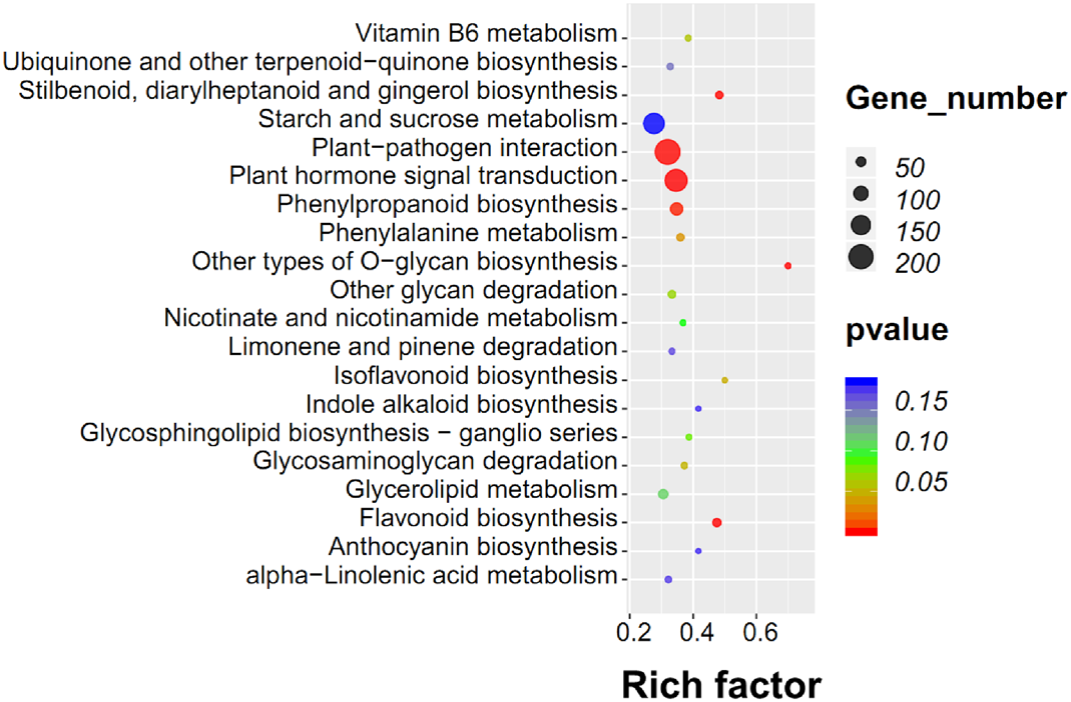
KEGG pathway enrichment analysis of DEGs. The x-axis represents the enrichment factor, while the y-axis represents the enrichment pathway. The dot sizes represent the number of differentially enriched genes. The statistical analysis of the pathway enrichment was performed using a Fisher’s exact test.

To further explore the biological functions of the DEGs, an enrichment analysis based on the KEGG database was performed. Among the 130 enriched KEGG pathways (Table S5), the strongest significance (*p* < 0.05) was observed for “plant hormone signal transduction” (*p* = 4.252E−06), “flavonoid biosynthesis” (*p* = 2.641E−05), “other types of O-glycan biosynthesis” (*p* = 4.647E−05), “stilbenoid, diarylheptanoid, and gingerol biosynthesis” (*p* = 2.110E−04), “plant-pathogen interaction” (*p* = 2.322E−04), “phenylpropanoid biosynthesis” (*p* = 1.778E−03), “phenylalanine metabolism” (*p* = 3.518E−02), and “isoflavonoid biosynthesis” (*p* = 4.660E−02) (Fig. 5).

### DEGs expression profiles associated with flavonoid biosynthesis

**Figure 6:**
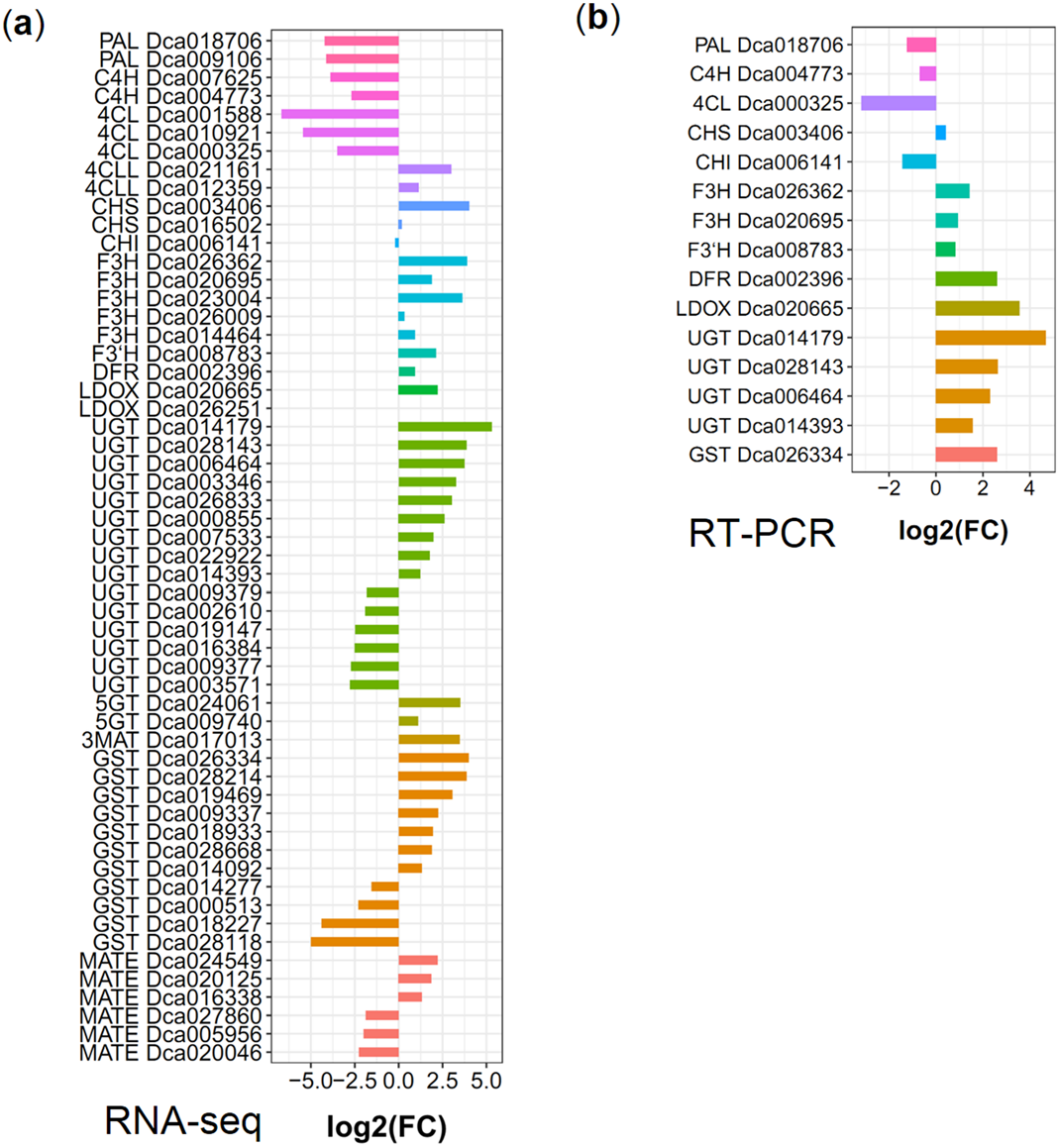
Relative expression levels of flavonoid biosynthesis-related structural genes in CK and Pr by RNA-seq (**a**) and RT-PCR (**b**). *PAL*: *phenylalanine ammonialyase; C4H*: *Cinnamate 4*-*hydroxylase*; *4CL*: *4-coumaroyl*: *CoA ligase*; *4CLL*: *4-coumaroyl*: *CoA ligase*-*like; CHS*: *chalcone synthase*; *CHI: chalcone isomerase*; *F3H*: *flavanone 3-hydroxylase*; *F3′H*: *Flavonoid 3′-hydroxylase; DFR*: *dihydroflavonol-4-reductase*; *LDOX*: *leucoanthocyanidin dioxygenase*; *3MAT*: *Anthocyanin 3-O-glucoside-6*″*-O-malonyltransferase*; *GST*: *glutathione S-transferase*; *MATE*: *Multidrug and Toxic Compound Extrusion* transporter families.

Flavonoids are synthesized through the phenylpropanoid metabolism pathway, and many enzymes participate in the catalyzing steps. In this study, the transcript levels of 56 flavonoid biosynthesis-related structural genes were analyzed (Fig. 6a). Two putative *PAL* (*Dca018706* and *Dca009106*) and two *C4H* (*Dca007625* and *Dca004773*) genes were identified, and all were down-regulated in Pr compared to CK. Five *4CL* (*Dca001588*, *Dca010921 ,Dca000325*, *Dca021161,* and *Dca012359*) genes were identified, among which the expression levels of *Dca021161* and *Dca012359* increased 8-fold and 2.2-fold in Pr vs. CK, respectively. Chalcone synthase (CHS) plays an important role in flavonoid biosynthesis; two putative *CHS* genes (*Dca003406* and *Dca016502*) were identified and *Dca003406* was up-regulated by 16.2-fold in Pr compared to in CK. One *CHI* (*Dca006141*) gene was identified and showed no change in Pr vs. CK. Expression of most of the five *F3H* genes were significantly increased in Pr vs. CK, including 14.8-fold *Dca026362,* 3.7-fold *Dca020695,* and 12.5-fold *Dca023004.* Expression of one *F3′H* (*Dca008783*) gene and one *DFR* (*Dca002396*) gene increased 4.4-fold and 1.9-fold, respectively. Two *LDOX* (*Dca020665* and *Dca026251*) were also identified, and *Dca020665* was up-regulated 4.7-fold in Pr vs. CK.

Usually, flavonoids are glycosylated in plant cells, thereby increasing their solubility and facilitating their transport^12^. Fifteen *UGT* genes were identified, and three genes showed more than tenfold up-regulation in Pr vs. CK, including 40-fold *Dca014179,* 14.8-fold *Dca028143,* and 13.4-fold *Dca006464.* Two *5GT* (*Dca024061* and *Dca009740*) and one *3MAT* (*Dca017013*) gene showed more than 11-fold upregulation in Pr compared to CK, except for *Dca009740* (Fig. 6a). Anthocyanins are synthesized on the cytosolic surface of the endoplasmic reticulum (ER) and transported to the vacuole^19^. Eleven *GST* genes and six *MATE* genes were identified, and most of these genes were upregulated in Pr vs. CK, such as 15.8-fold *Dca026334,* 14.7-fold *Dca028214,* 8.3-fold *Dca019469*, and 4.6-fold *Dca024549* (Fig. 6a). Moreover, 15 anthocyanin biosynthesis-related genes were chosen for expression validation by qRT-PCR (Fig. 6b, Figure S7). All of these 15 selected genes showed similar expression patterns in the RNA-seq data, which indicated that the RNA-seq data were reliable.

### Integrated metabolomic and transcriptomic analyses in phenylpropanoid metabolism

**Figure 7:**
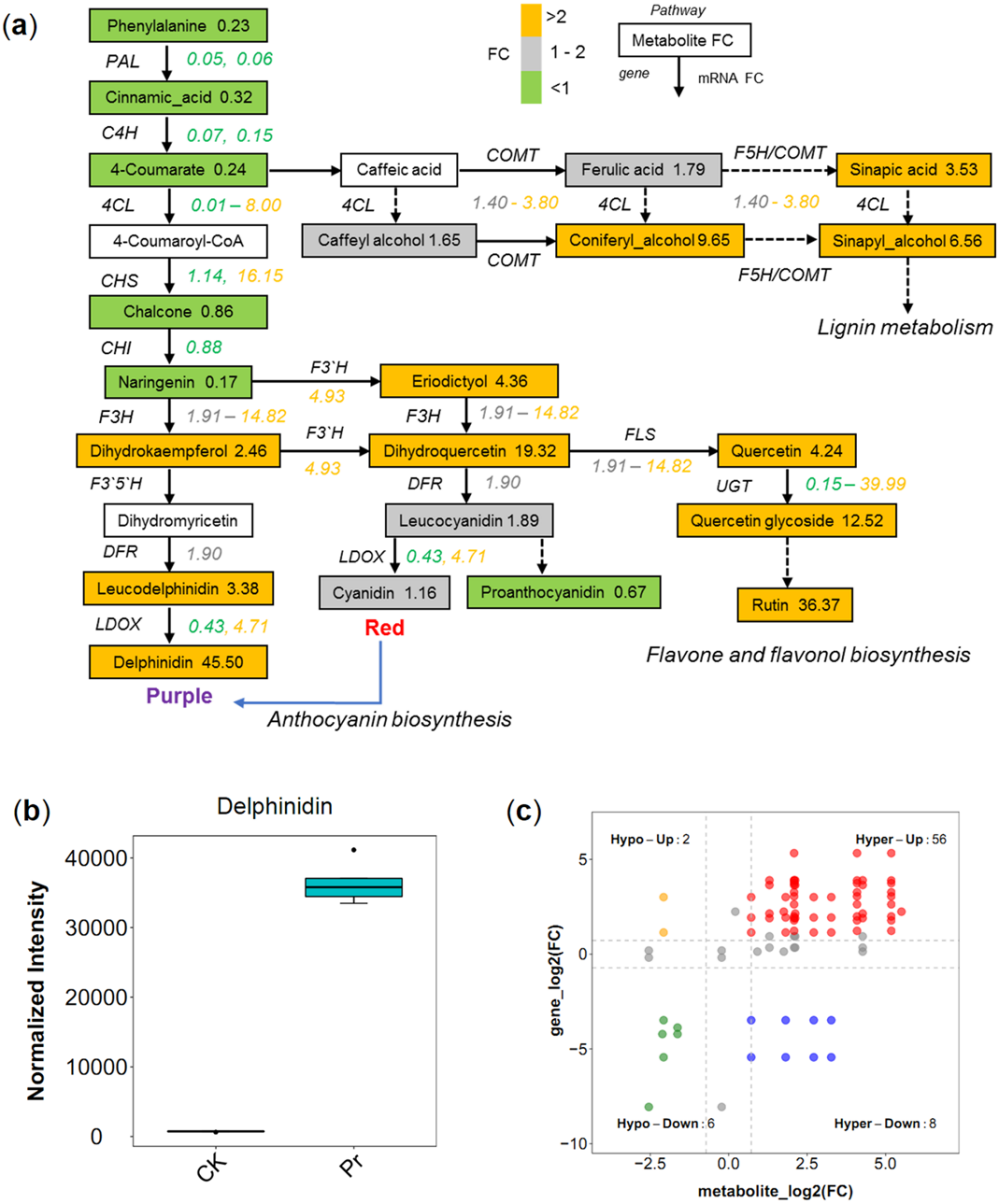
Mapping the phenylpropanoid pathway in *D. officinale*. (**a**) Simplified metabolic flow charts describing changes in metabolites, related mRNAs of Pr vs. CK. FCs in mRNA (underlined on right) and metabolites levels (in box) in Pr vs. CK are listed and highlighted by color. Compartments with no color/no values indicate metabolites/mRNAs that were not detected. (**b**) Delphinidin content in Pr and CK. The error bars show the maximum and minimum of the distributions and circles identify outlying data points (*n* = 6 biologically independent samples). (**c**) Quadrant diagrams representing association of metabolomic and transcriptomic variation. The color dots indicate metabolites and/or mRNAs whose abundances were impacted > 1.2-fold and *p*-value < 0.05. The metabolites and mRNAs are in red and green dots, are positively correlated and have similar consistent patterns, while the metabolites and mRNA shown in orange and blue dots, are negatively correlated and have opposite patterns. Unchanged metabolites and unchanged genes are displayed as gray dots.

We integrated metabolomic and transcriptomic data to analyze the phenylpropanoid metabolism pathway in *D. officinale* (Fig. 7a). The phenylpropanoid pathway initiates from the aromatic amino acid phenylalanine, which decreased 4.3-fold in Pr vs. CK. Cinnamic acid decreased 3.1-fold in Pr vs. CK, while two *PAL* were down-regulated by 18.7-fold and 17.3-fold, respectively. 4-Coumarate decreased 4.2-fold in Pr vs. CK, while two *C4H* were down-regulated by 14.7-fold and 6.5-fold, respectively. Two *CHS* were up-regulated by 16.2-fold and 1.1-fold, respectively; nevertheless, chalcone decreased 1.2-fold in Pr vs. CK. Naringenin decreased 5.8-fold in Pr vs. CK, which was sustained by a 1.1-fold decrease in its mRNA. The 4CL family plays key roles in lignin metabolism and participates in sinapoyl-CoA, feruloyl-CoA, and caffeoyl-CoA biosynthesis^12^. We found that sinapic acid, conifer alcohol, and sinapoyl alcohol increased 3.53-fold, 9.65-fold, and 6.56-fold, respectively. One *COMT* gene (*caffeic acid 3-O-methyltransferase*) increased 3.80-fold in Pr compared to CK. Lignan is also an important active compound in Chinese herbs, which shares a common upstream pathway with lignins and branches after the synthesis of coniferyl alcohol^30^. These results suggest that the medicinal value of *D. officinale* requires further exploration.

As with lignin metabolism, increased levels of flavone and flavonol (i.e., dihydrokaempferol, dihydroquercetin, eriodictyol, quercetin, quercetin glucoside, and rutin) were associated with significant increases in mRNA (Fig. 7a). For example, dihydroquercetin was estimated to increase 19-fold in Pr compared to that in CK, which was underpinned by a fivefold increase in its mRNA. Substantial changes in Pr were also observed for compounds connected to anthocyanin metabolism (Fig. 7a). Anthocyanin metabolism plays an important role in plant coloration; cyanidin and delphinidin contribute to red to magenta and magenta to purple colors, respectively^12^. In Pr the total delphinidin content increased 45 times compared to CK, while one *LDOX* (*Dca020665*) was up-regulated 4.7-fold and the other (*Dca026251*) showed no obvious change, suggesting that the pigmentation of Pr was due to delphinidin (Fig. 7a,b).

For a systematic overview of the associations between the DAMs and DEGs, we compared the log_2_ fold changes between Pr and CK in mRNA and metabolites in Fig. 7c (the data details are shown in Table S8, Table S9). The metabolites and their correlated mRNA shown in red dots and green dots were both more abundant in Pr. For instance, three up-regulated genes (*Dca026362, Dca020695,* and *Dca023004*) of *F3H* were correlated with accumulation of dihydrokaempferol, dihydroquercetin, quercetin and eriodictyol. Down-regulated *PAL* (*Dca018706*) was correlated with the reduction of phenylalanine and cinnamic acid. The mRNA shown in orange dots, were more abundant than metabolites in Pr, while the metabolites, shown in blue dots, were more abundant than mRNA in Pr. For instance, two *4CLL* (*Dca021161*, *Dca012359*) were up-regulated and correlated with the reduction of 4-coumarate. The other two *4CL* (*Dca010921* and *Dca000325*) were down-regulated and correlated with the accumulation of caffeyl alcohol, coniferyl alcohol, sinapyl alcohol and sinapic acid.

### Hormone signal transduction pathway associated with flavonoid biosynthesis

**Figure 8:**
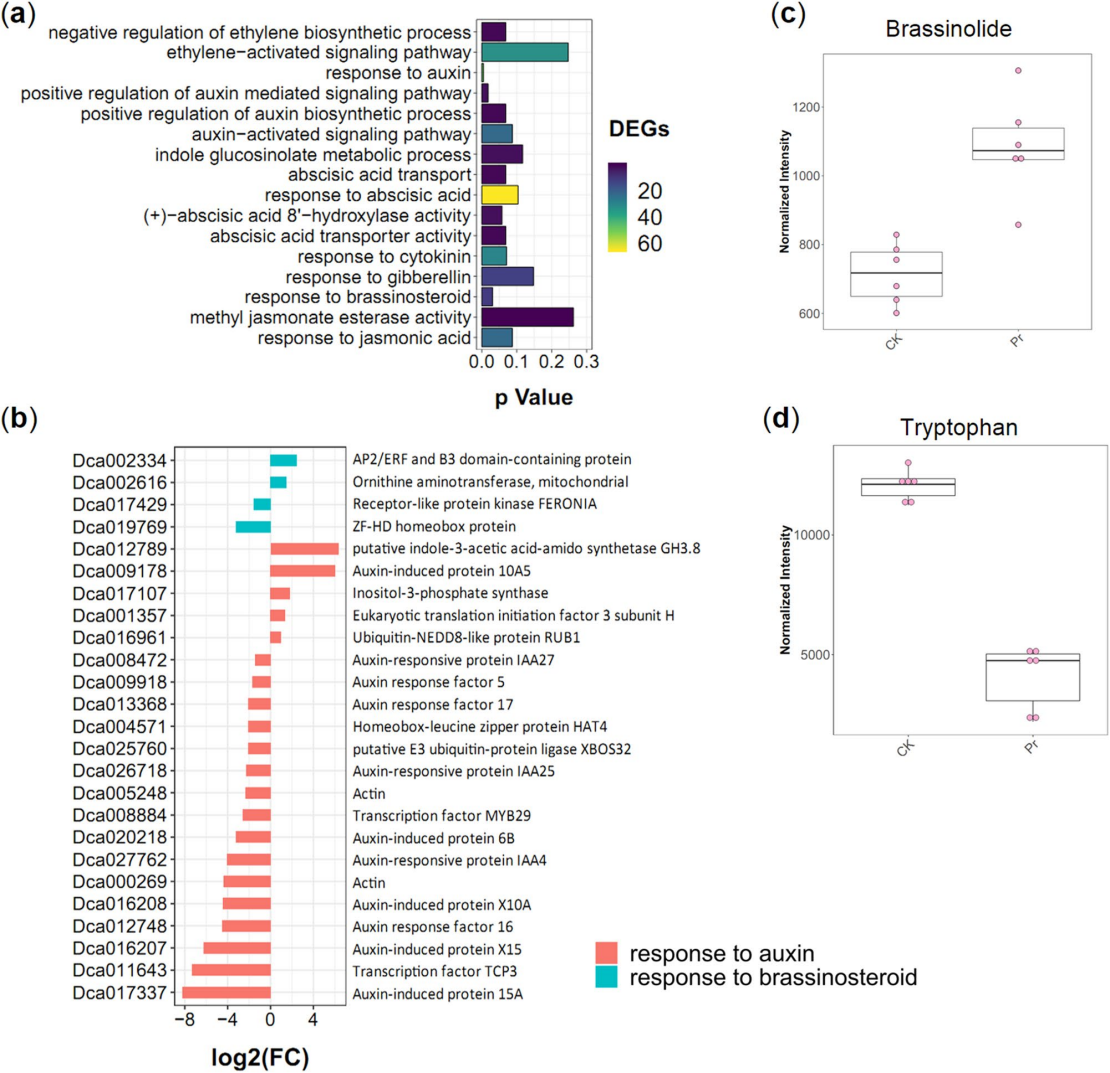
Hormone signal transduction pathway associated with flavonoid biosynthesis. (**a**) Enrichment analysis of 16 phytohormones-related GO terms. (**b**) Expression analysis of genes related to auxin and brassinosteroid. (**c**) Brassinolide content in Pr and CK. (**d**) Tryptophan content in Pr and CK. The error bars show the maximum and minimum of the distributions and circles identify outlying data points (*n* = 6 biologically independent samples).

Based on the transcriptomes, 106 significant DEGs were enriched in “Plant hormone signal transduction” (ko04075), most of which are involved in the auxin, cytokinin, ethylene, JA, ABA, GA, SA, and BR signaling pathways (Figure S8, Table S6). To further explore plant hormone metabolism associated with flavonoid biosynthesis, 16 related GO terms were identified, and two pathways were significantly enriched (*p* < 0.05) in Pr compared to in CK (Fig. 8a), including “response to auxin” (GO:0009733), “positive regulation of auxin-mediated signaling pathway” (GO:0010929), and “response to brassinosteroid” (GO:0009741). For the GO term “response to auxin”, we analyzed the expression of 21 auxin metabolism- and signaling pathway-related genes and found that most of the genes were down-regulated in Pr (Fig. 8b). In “response to brassinosteroid”, genes encoding AP2/ERF and B3 domain-containing protein and ornithine aminotransferase (*Dca002334* and *Dca002616*) were significantly up-regulated in Pr, while two other genes (*Dca017429* and *Dca019769*) were down-regulated in Pr (Fig. 7b). The endogenous brassinolide content was higher in Pr than in CK (Fig. 8c), and the auxin precursor (tryptophan) content was reduced in Pr (Fig. 8d). Together, these results suggest that BR and auxin metabolism affect flavonoid accumulation in Pr.

### Identifications of TFs families in Pr vs. CK

**Figure 9:**
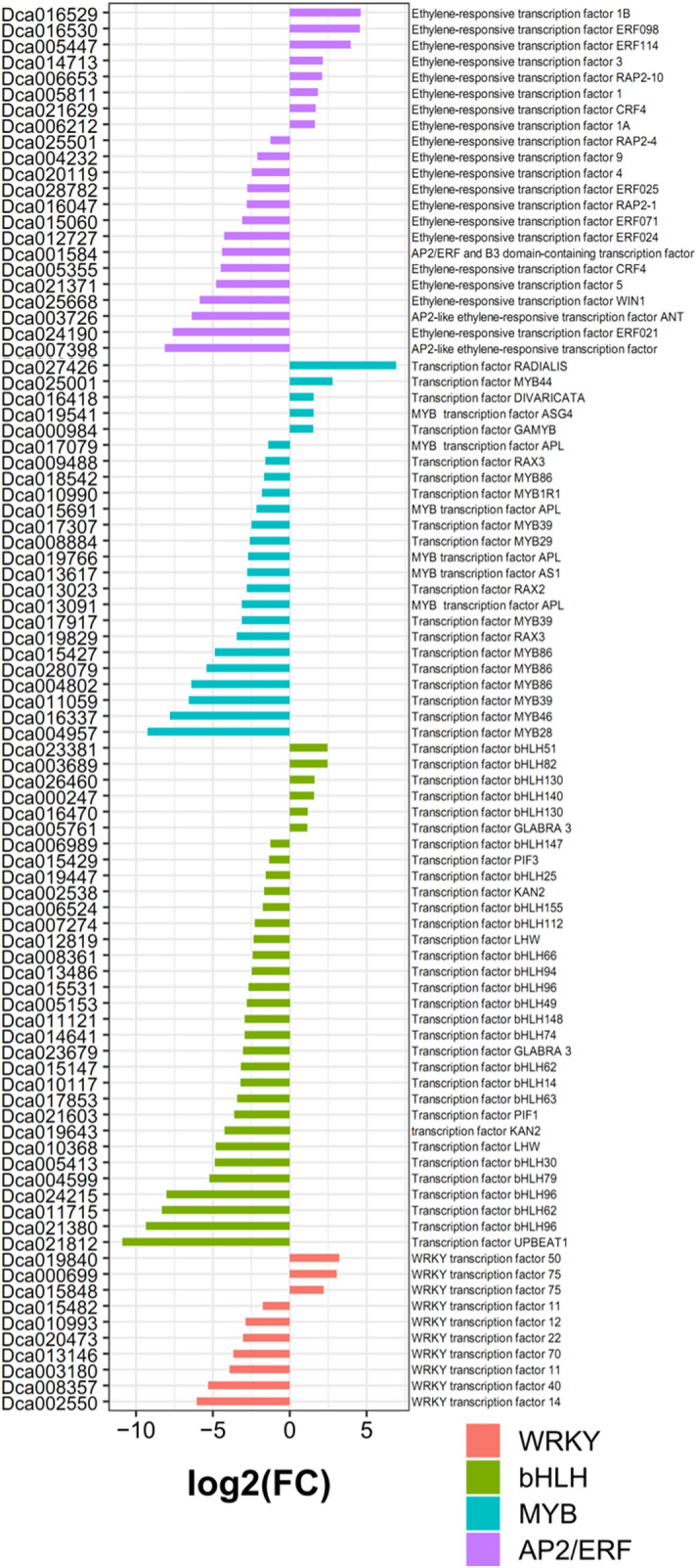
Relative expression levels of WRKY, bHLH, MYB and AP2/ERF in CK and Pr.

Several TFs have been reported to play important roles in flavonoid biosynthesis. In our study, 477 putative TF encoding genes belonging to 16 major TF families were analyzed in *D. officinale* (Table S10). A large number of TFs were included in the MYB family (90 genes), bHLH family (85 genes), AP2/ERF family (78 genes) and WRKY family (54 genes) (Table S11). Furthermore, DEGs analysis revealed that most of the TFs were significantly down-regulated in Pr (Fig. 9). For example, the AP2/ERF family, *Dca024190* and *Dca007398*, decreased 118-fold and 141-fold in Pr vs. CK, respectively. The MYB family (*Dca004957*) was greatly decreased by 434-fold in Pr vs. CK. Three bHLH family (*Dca024215*, *Dca011715*, and *Dca021380*) were down-regulated by more than 100-fold in Pr vs. CK. However, some up-regulated TFs showed especially strong responses in Pr (Fig. 9). For example, the AP2/ERF family, *Dca016529* and *Dca016530* were more than 20-fold up-regulated in Pr vs. CK. The MYB-related transcription factor RADIALIS (*Dca027426*) was greatly increased by 119-fold in Pr vs. CK.

## Discussion

Polysaccharides and alkaloids are common active constituents of *Dendrobium* species^31–33^. The biosynthesis of polysaccharides and alkaloids is strongly associated with plant primary and secondary metabolism, and plants contain three major groups of secondary metabolites: phenolics, terpenoids, and alkaloids. Phenylpropanoids are the first class of phenolics that can be divided into several groups, such as flavonoids, anthocyanins, and lignin^34^. However, few studies have qualitatively or quantitatively studied phenylpropanoids in *Dendrobium*. Eight flavonoid glycosides were identified in *D. catenatum* from three locations. Total flavonoid content in Guangxi samples were the highest, with a content of 3.87 μg/mg, while the total flavonoid content of the samples from the Guangdong and Zhejiang provinces were lower, at 2.40 and 2.85 μg/mg, respectively^8^. In this study, we identified a Pr of *D. officinale* with higher flavonoid and anthocyanin content in stems compared to that in CK (Fig. 1). Nontargeted metabolomics revealed 148 different metabolites involved in the biosynthesis of phenylpropanoids, amino acids, purines, and organic acids (Fig. 2). Phenylpropanoid biosynthesis is a complex process, and the initial three steps of the pathway, catalyzed by PAL, C4H, and 4CL, are necessary and provide the basis for all subsequent branches^12^. This suggests that the *PAL*, *C4H,* and *4CL* genes play important roles in flavonoid enrichment. The *pal1 pal2* double mutants were deficient of tannin pigments in the seed coat^35^. However, two isoforms of *PAL* and *C4H* were identified in our transcript data, and both were down-regulated in Pr compared to CK (Fig. 6). It could be that the late biosynthetic genes play a more important role than the early biosynthetic genes in phenylpropanoid metabolism in mature plants. For instance, the overexpression of *SQD2.2*, which encodes a glycosyltransferase in rice, conferred plants with enhanced flavonoid levels at the mature stage^36^. Flavonol synthase (FLS) and dihydroflavonol 4-reductase (DFR) have strong competition for common dihydromyricetin substrates that affect flower color in grape hyacinth^37^.

Integrated transcriptomic and metabolomic analyses demonstrated the gene-to-metabolite networks and enabled the mechanisms involved in Pr pigmentation to be deciphered (Fig. 7a). The flow of individual metabolites with mRNA, including *F3H*, *DFR*, and *LDOX*, suggests that delphinidin is the predominant anthocyanin in the purple variety. The biosynthesis of anthocyanin via the phenylpropanoid pathway is controlled by two types of genes: structural genes and regulatory genes. Structural genes can be divided into early biosynthetic genes (such as *PAL* and *C4H*) and late biosynthetic genes (such as *LDOX* and *UGT*)^14^. In our study, the expression pattern of late biosynthetic genes was more complex than that of late biosynthetic genes. For example, *PAL* and *C4H* correlated with their metabolites and were down-regulated in Pr vs. CK. In contrast, one *LDOX* (*Dca020665*) was up-regulated 4.7-fold and the other (*Dca026251*) showed no obvious change in Pr vs. CK, and their related products, delphinidin and cyanidin, increased by 45.5-fold and 1.1.6-fold, respectively, in Pr vs. CK (Fig. 7a). Regulatory genes including MYB, bHLH and WD-repeat proteins, constitute a ternary complex predominantly responsible for orchestrating regulation of anthocyanin synthesis^38^. Our transcriptomic data identified 24 MYB genes and 32 bHLH genes as DEGs in Pr vs. CK, and most of them were significantly down-regulated (Fig. 9). This could be due to metabolic feedback or the preference of regulatory genes. In Arabidopsis, the MYB-bHLH-WD40 complex predominantly regulates late biosynthetic genes over early biosynthetic genes^39^. The expression of other late biosynthetic genes, such as *DFR* and *LDOX*, is nearly off or is undetectable in *ttg1* and bHLH anthocyanin mutants^39^. Moreover, anthocyanin accumulation is closely related to subcellular transport, including vesicle trafficking, membrane transporters and GST^20^. GSTs are also associated with ER and vacuole membranes that produce high levels of anthocyanins^40,41^. In Arabidopsis, the Golgi-localized membrane protein can transport proanthocyanidin to the central vacuole via vesicle trafficking^42^. In our study, a large number of DEGs were classified into eight GO terms related to “cellular component”, such as “plasma membrane” and “trans-Golgi network” (Fig. 4b). This finding suggests that membrane transport is likely to be an important regulator of anthocyanin accumulation in Pr.

The final flower color is determined by three major anthocyanins: pelargonidin, cyanidin, and delphinidin. Cyanidin contributes to red to magenta colors, and delphinidin contributes to magenta to purple colors^12^. Anthocyanins are widely present in plants and are responsible for the purple coloration of plant stems and leaves. Several studies have reported that sugars (e.g., glucose, sucrose, maltose, and turanose) induce anthocyanin biosynthesis in Arabidopsis hypocotyls and leaves^43,44^. The stems of cultivated *D. officinale* are rich in mannose and glucose in the mature stage^45^, suggesting that the purple variety of *D. officinale* is a good model for studying the regulatory mechanisms of secondary metabolite accumulation. Dihydrokaempferol and dihydroquercetin provided substrates for quercetin derivative biosynthesis that were significantly up-regulated in Pr, especially rutin (Fig. 7a). Rutin is a potent antioxidant that inhibits sorbitol, reactive oxygen species, advanced glycation end-product precursors, and inflammatory cytokines^46^. Therefore, the active medicinal components of *D. officinale* require further exploration for their pharmacological potential.

Auxin is an essential hormone that regulates almost every aspect of plant growth and development. Normally, auxin is transported from synthesis sites in the apices to the distal parts of the plant. Indole-3-acetic acid (IAA) is the primary natural auxin that plays a role in gravity-induced flavonoid synthesis in the root tips^21^. IAA is synthesized via tryptophan (Trp)-dependent and Trp-independent pathways^47^. Our data showed that the Trp content was reduced in Pr (Fig. 8d) and that most of the auxin-related genes were down-regulated in Pr (Fig. 8b). In Arabidopsis *tt* mutants, the flavonol-overproducing mutants exhibited reduced auxin transport, whereas the no flavonoids-accumulating mutant increased auxin transport^48–50^. These data suggest that high flavonoid accumulation is negatively correlated with auxin metabolism in Pr. Brassinolide, the most potent BR, is produced by several cytochrome P450 monooxygenases from campesterol^51^. Similar to animal hormones, acetyl-CoA enters the mevalonic acid pathway to make mevalonate^26^. Some studies have indicated that exogenous BR could stimulate the biosynthesis of flavonoids in leaves^52^. Moreover, brassinolide signaling has been implicated in PIN2 sorting and intracellular distribution to delimit root gravitropism^53^. Our transcriptomic data identified the “response to brassinosteroid” and “lipid and wax metabolism” GO terms, and the endogenous brassinolide contents were increased in Pr compared to CK (Fig. 8c, Figure S6). Therefore, brassinolide stimulated increased flavonoid concentrations in Pr. These results suggest that brassinolide signaling and auxin signaling crosstalk play important roles in the regulatory network of phenylpropanoid metabolism in *D. officinale.*

## Materials and methods

### Plant materials and culture conditions

The stem samples were harvested from two-year-old cultivated *D. officinale,* including the purple variety and normal variety, in August 2019 from a growth chamber of Zhejiang University, Hangzhou, China. The growth conditions were set at 25 ± 2 °C with a light/dark cycle of 12/12 h and a 65–75% relative humidity. Stem tissues were collected from three independent plants for transcriptomic analysis (three biological replicates). The stem tissues were collected from six independent plants for metabolome analysis (six biological replicates). All samples were immediately frozen in liquid nitrogen and stored at − 80 °C.

### Nontargeted metabolomic and analysis

The collected samples were thawed on ice, and metabolites were extracted with 50% methanol buffer^54^. Briefly, 20 μL of sample was extracted with 120 μL of precooled 50% methanol, vortexed for 1 min, and incubated at room temperature (24 ± 2 °C) for 10 min. The samples were stored at − 80 °C prior to the LC–MS analysis. In addition, pooled QC samples were prepared by combining 10 μL of each extraction mixture.

For metabolite separation, we used the method previously described by Dunn et al.^54^. First, all chromatographic separations were performed using an ultra-performance liquid chromatography (UPLC) system (SCIEX, UK). An ACQUITY UPLC T3 column (100 mm × 2.1 mm, 1.8 µm, Waters, UK) was used for the reversed phase separation. The column oven was maintained at 35 °C. The flow rate was 0.4 mL/min and the mobile phase consisted of solvent A (water, 0.1% formic acid) and solvent B (Acetonitrile, 0.1% formic acid). Gradient elution conditions were set as follows: 0–0.5 min, 5% B; 0.5–7 min, 5% to 100% B; 7–8 min, 100% B; 8–8.1 min, 100% to 5% B; 8.1–10 min, 5% B. The injection volume for each sample was 4 µL.

A high-resolution tandem mass spectrometer TripleTOF 5600plus (SCIEX, UK) was used to detect metabolites eluted from the column^54^. The Q-TOF was operated in both positive and negative ion modes. The curtain gas was set 30 PSI, ion source gas1 was set 60 PSI, ion source gas2 was set 60 PSI, and ion source temperature was 650 °C. For the positive ion mode, the ions pray voltage floating was set at 5000 V. For the negative ion mode, the ions pray voltage floating was set at 4500 V. The mass spectrometry data were acquired in IDA mode. The TOF mass ranged from 60 to 1200 Da. The survey scans were acquired in 150 ms and as many as 12 product ion scans were collected if a threshold of 100 counts per second (counts/s) was exceeded with a 1 + charge state. The total cycle time was fixed to 0.56 s. Four time bins were summed for each scan at a pulser frequency value of 11 kHz by monitoring the 40 GHz multichannel TDC detector with four-anode/channel detection. Dynamic exclusion was performed for 4 s. During the acquisition, the mass accuracy was calibrated for every sample. The online KEGG database was used to annotate the metabolites by matching the exact molecular mass data (m/z) of samples with those from the database. If the mass difference between the observed and the database value was less than 10 ppm, the metabolite would be annotated and the molecular formula of metabolites would further be identified and validated by the isotopic distribution measurements. We also used an in-house fragment spectrum library of metabolites to validate metabolite identification. Student’s *t*-tests were conducted to detect differences in metabolite concentrations between the two phenotypes. The *p*-value was adjusted for multiple tests using the FDR (Benjamini–Hochberg). Supervised PLS-DA was conducted through metaX to discriminate the different variables between groups^55^. Metabolites with significant differences in content were defined as having a variable importance in the project (VIP) ≥ 1 and a fold change of ≥ 2 or ≤ 0.5.

### Flavonoid and anthocyanin measurement

Flavonoids were washed twice with 70% ethanol to remove chlorophyll, and the remaining sediment was suspended in methanol (1:10, w/v) and incubated overnight in the dark at 4 °C to extract the flavonoids. The methanol extract was used to measure the concentration of total flavonoids, which was determined using the AlCl_3_ method as described previously^52^. Absorbance at 510 nm for flavonoids was determined, and rutin was used as the standard.

Anthocyanin content of the stem was determined using the protocol described by Mita et al.^56^. The stems (100 mg) were extracted for 1 day at 4 °C in 1 mL of 1% (v/v) hydrochloric acid in methanol. The mixture was centrifuged at 13,000 rpm for 15 min and the absorbance of the supernatant was measured at 530 nm and 650 nm.

Thin layer chromatography of all extracts was performed on TLC precoated silica gel 60 GF254 plate using hexane ethyl acetate formic acid (7:3:0.1) as solvent system. TLC plates were detected under UV light at 254 nm and 366 nm. TLC fingerprints were determined using the protocol outlined by Sithisarn et al.^57^.

### Transcriptomic analysis

Stem samples from three independent biological replicates for both CK and Pr were harvested and frozen in liquid nitrogen. Total RNA was extracted using Trizol reagent (Invitrogen, CA, USA) following the manufacturer's procedure^32^. Total RNA quantity and purity were analyzed with Bioanalyzer 2100 and RNA 6000 Nano LabChip Kit (Agilent, CA, USA) with RIN number > 7.0. Approximately 10 µg of total RNA representing a specific adipose type was subjected to isolation of (A) mRNA with poly-T oligo attached magnetic beads (Invitrogen). Following purification, the mRNA was fragmented into small pieces using divalent cations at elevated temperatures. Then, the cleaved RNA fragments were reverse-transcribed to create the final cDNA library in accordance with the protocol for the mRNASeq sample preparation kit (Illumina, San Diego, USA).The average insert size for the paired-end libraries was 300 bp (± 50 bp). Paired-end sequencing was performed on an IlluminaHiseq4000 (Illumina Inc., San Diego, CA, USA) by the Lianchuan Biotechnology Company (Hangzhou, China). The RNA-seq data were been submitted to the BIG Data Center of the Chinese Academy of Sciences (https://bigd.big.ac.cn) with accession number CRA002691.

### Differentially expressed genes analysis

The raw data were filtered using Trimmomatic (https://www.usadellab.org/cms/?page=trimmomatic) and then were assessed using FastQC (https://www.bioinformatics.babraham.ac.uk/projects/fastqc/) to obtain clean reads. Clean reads were aligned to the reference genome of *D. catenatum*^2^ using the HISAT program^58^. Subsequently, the expression levels of unigene were calculated as FPKM (fragments per kilo bases of exons for per million mapped reads) using the software package StringTie^59^. The DEGs were further characterized and estimated using the edgeR package according to the results from StringTie^60^. FDRs ≤ 0.05, log_2_ fold-change (FC) > 1 or log_2_ FC < − 1 and with statistical significance (*p* value < 0.05) were used as threshold for determining the DEGs between Pr and CK. The DEGs were also subjected to Gene Ontology (GO) enrichment analysis using GOseq software^61^ and KEGG pathway enrichment analysis by KOBAS 2.0^62^.

### Real-time quantitative PCR

Total RNA was isolated from different tissues using TransZol reagent (TransGen Biotech, Beijing). RNA extracts were treated with DNaseI (NEB, UK) to eliminate DNA contamination. First-strand cDNA was produced from the RNA template by reverse transcription using the TIANscriptRTKit according to the manufacturer's instructions (TransGen Biotech, Beijing). Quantitative real-time PCR analyses were performed using a SYBRGreen qPCRkit (TransGenBiotech) with a MyiQ system (Bio-Rad) as described previously^63^. The primers are listed in Table S7.

### Statistical analysis

The data are displayed as the mean ± standard deviation (SD). Statistical analyses were performed using SPSS 17.0 software (SPSS Inc., Chicago, IL USA), and an ANOVA was applied to compare the differences between the two groups. Data were also treated by hierarchical clustering using the R package pheatmap and by a principal component analysis (PCA) using the R package FactoMineR (R version 3.6.0, https://cran.r-project.org/bin/windows/base/old/3.6.0/).

## Supplementary information

Supplementary Tables.

Supplementary Figures.
