# Supplementary material for: Metabolomic and transcriptomic analyses reveal the regulation of pigmentation in the purple variety of Dendrobium officinale
Source: Sci Rep. 2020 Oct 19;10:17700. doi: 10.1038/s41598-020-74789-0 (PMC7573623; doi:10.1038/s41598-020-74789-0)
Supplement: Supplementary file 2 — Supplementary Figures. [file 41598_2020_74789_MOESM2_ESM.pptx]

## Slide 1
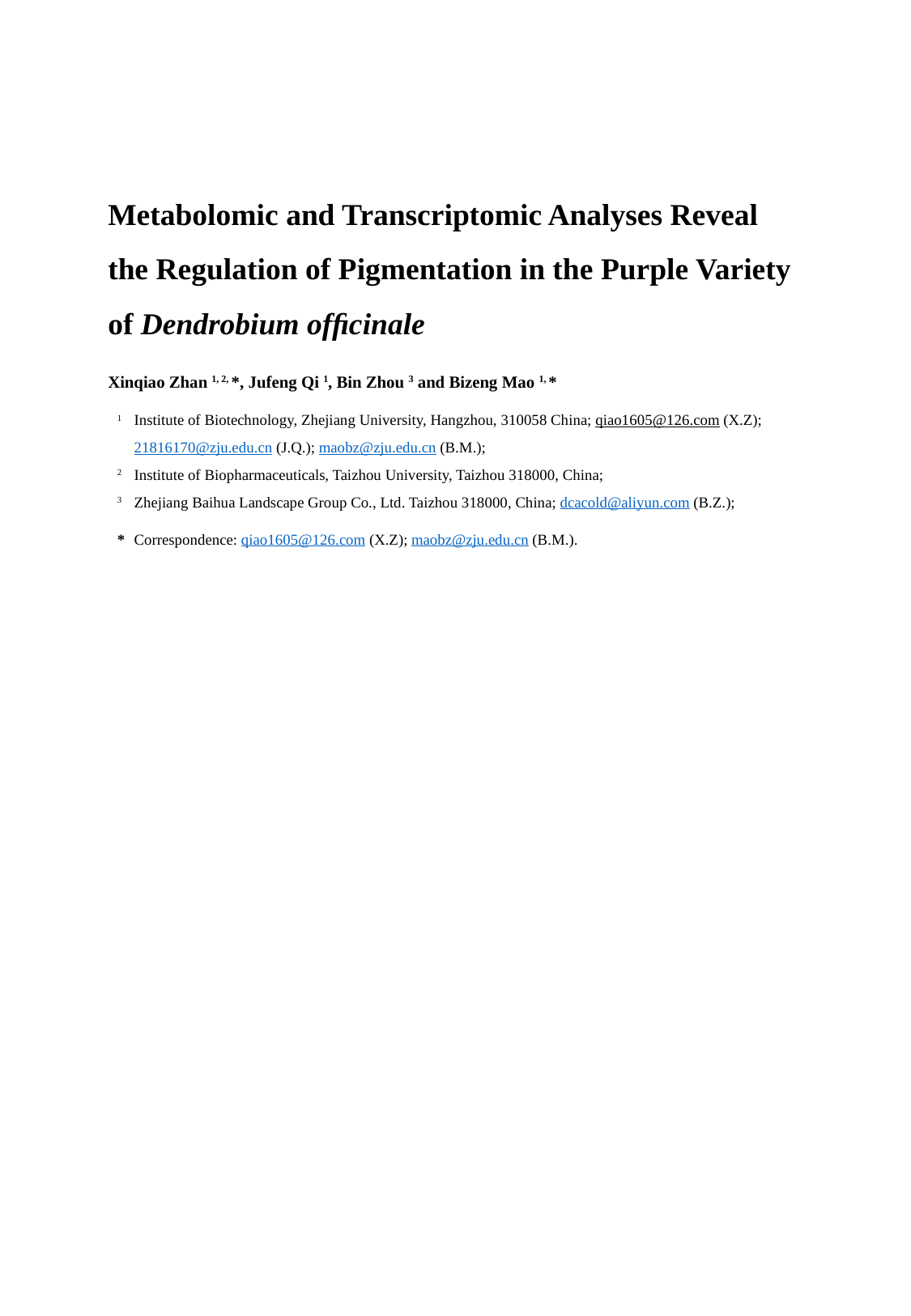

Metabolomic and Transcriptomic Analyses Reveal the Regulation of Pigmentation in the Purple Variety of Dendrobium ofﬁcinale
Xinqiao Zhan 1, 2, *, Jufeng Qi 1, Bin Zhou 3 and Bizeng Mao 1, *
1	Institute of Biotechnology, Zhejiang University, Hangzhou, 310058 China; qiao1605@126.com (X.Z); 21816170@zju.edu.cn (J.Q.); maobz@zju.edu.cn (B.M.);
2	Institute of Biopharmaceuticals, Taizhou University, Taizhou 318000, China;
3	Zhejiang Baihua Landscape Group Co., Ltd. Taizhou 318000, China; dcacold@aliyun.com (B.Z.);
*	Correspondence: qiao1605@126.com (X.Z); maobz@zju.edu.cn (B.M.).

## Slide 2
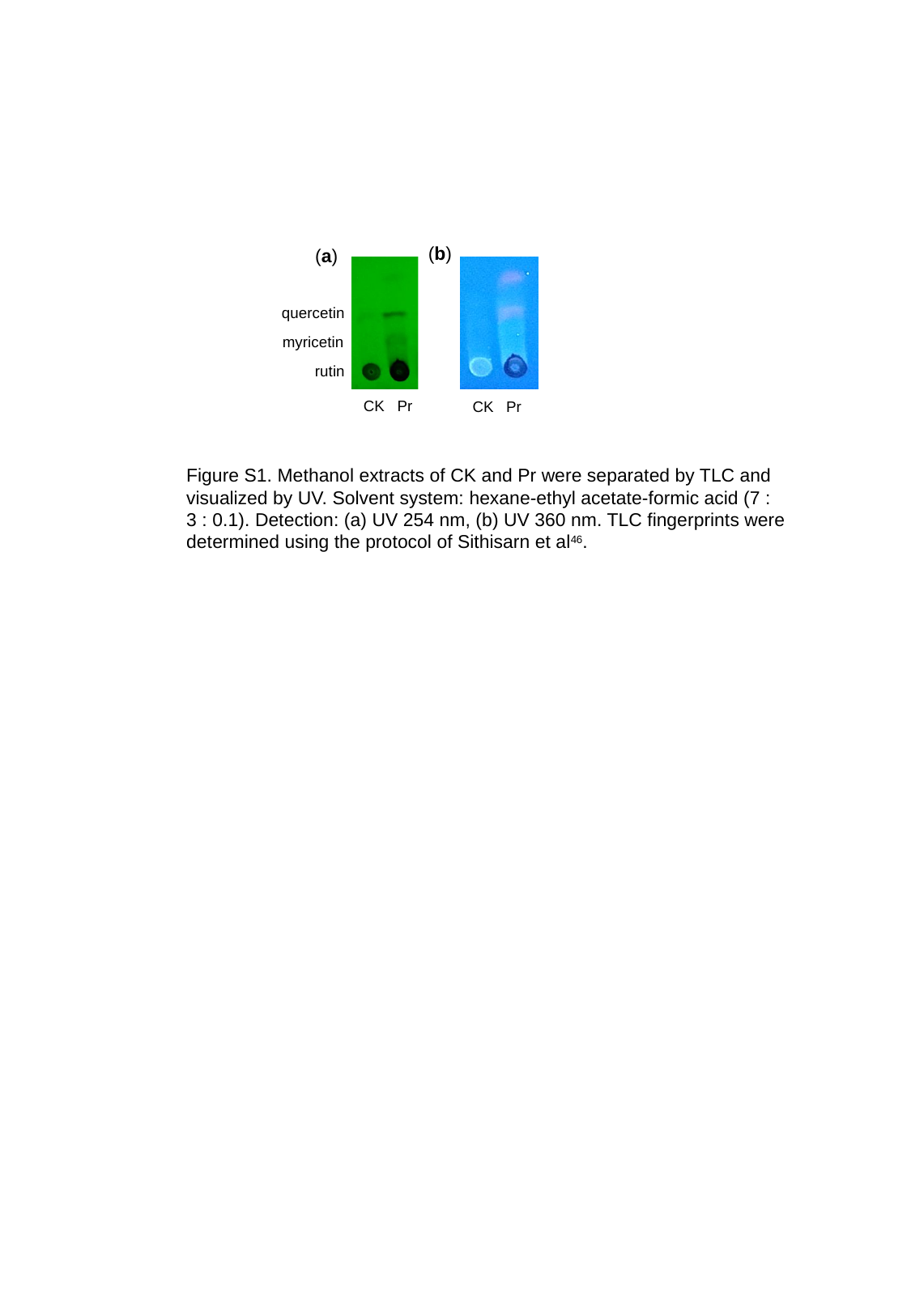

(b)
(a)
quercetin
myricetin
rutin
CK Pr
CK Pr
Figure S1. Methanol extracts of CK and Pr were separated by TLC and visualized by UV. Solvent system: hexane-ethyl acetate-formic acid (7 : 3 : 0.1). Detection: (a) UV 254 nm, (b) UV 360 nm. TLC fingerprints were determined using the protocol of Sithisarn et al46.

## Slide 3
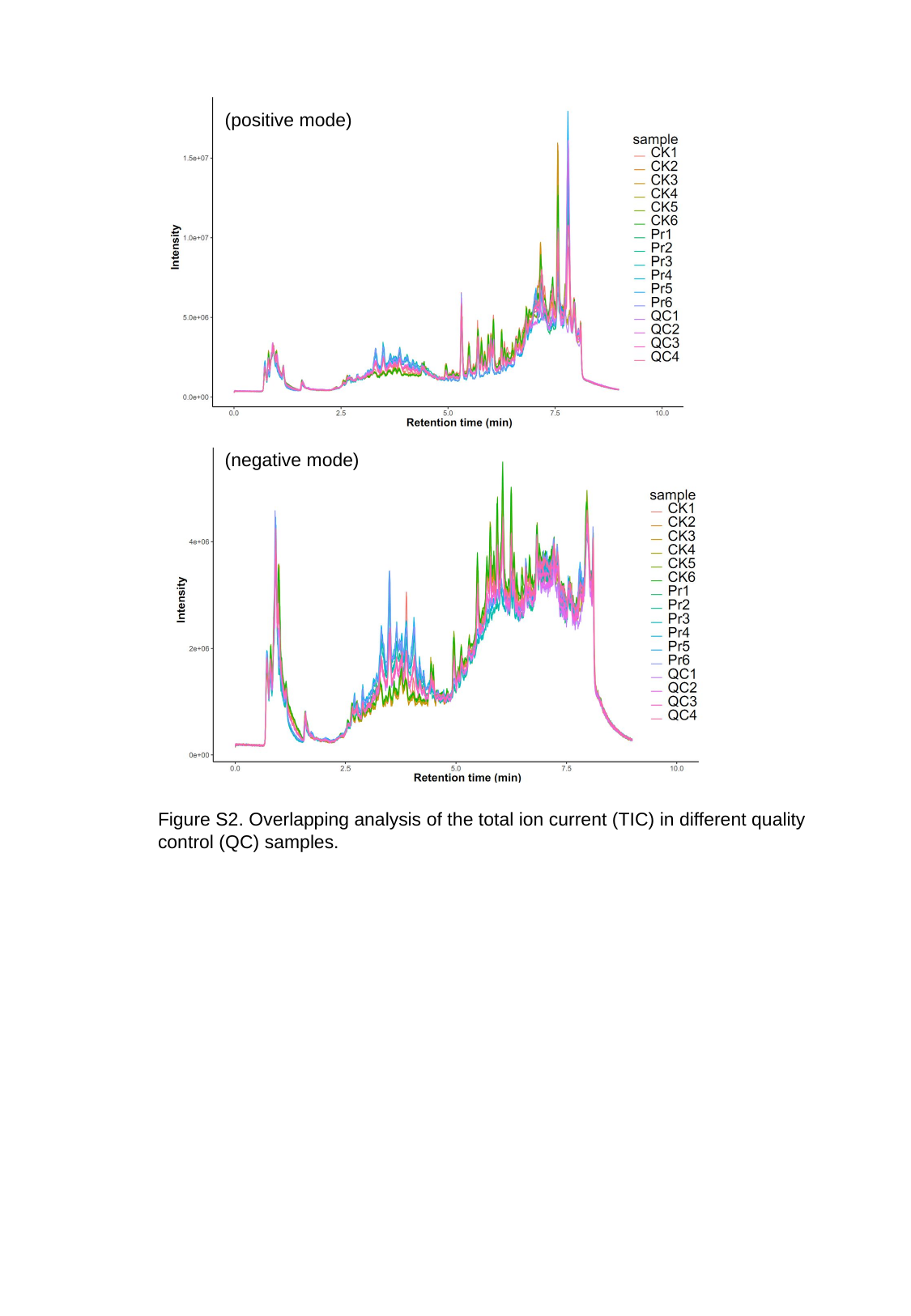

(positive mode)
(negative mode)
Figure S2. Overlapping analysis of the total ion current (TIC) in different quality control (QC) samples.

## Slide 4
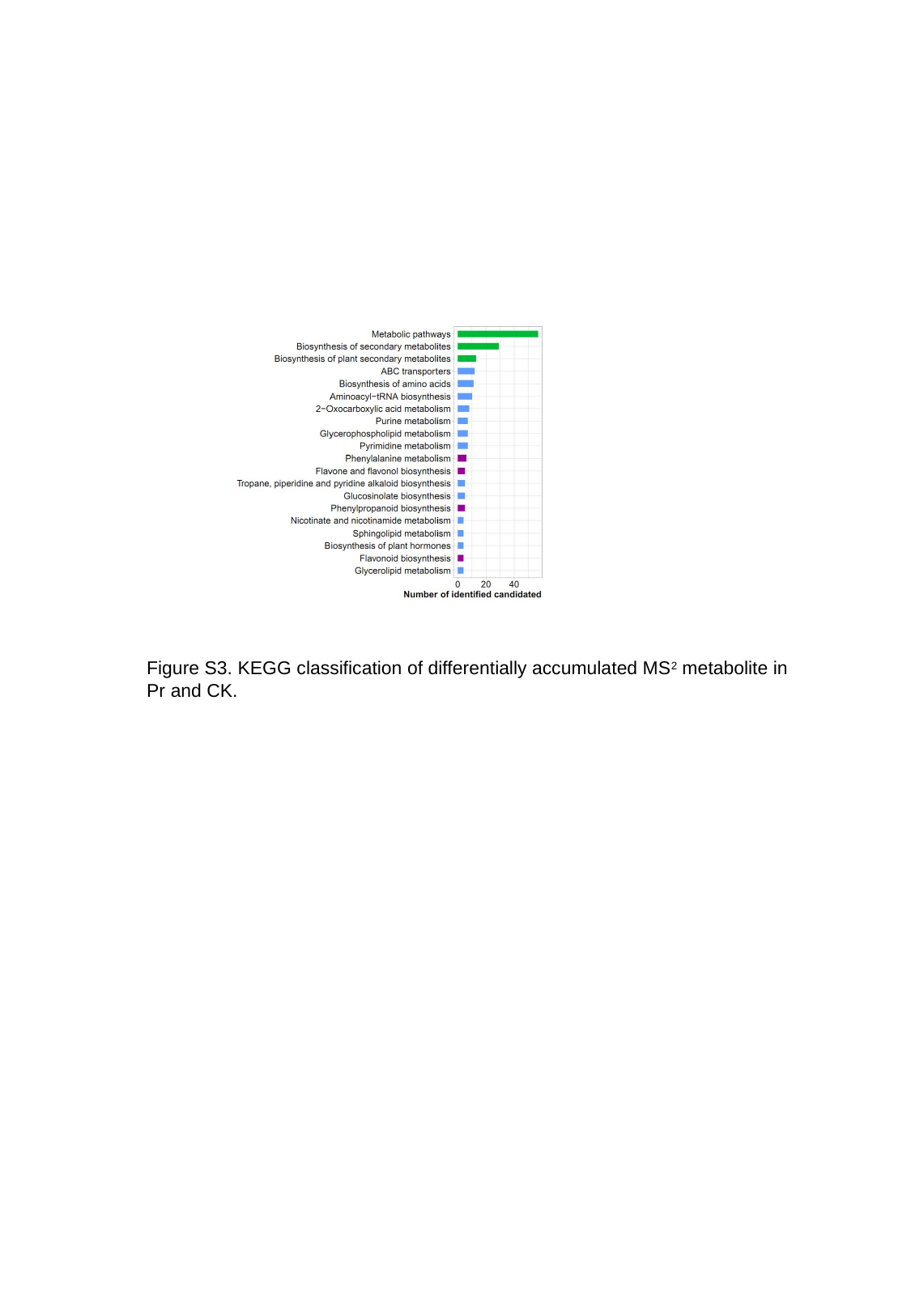

Figure S3. KEGG classification of differentially accumulated MS2 metabolite in Pr and CK.

## Slide 5
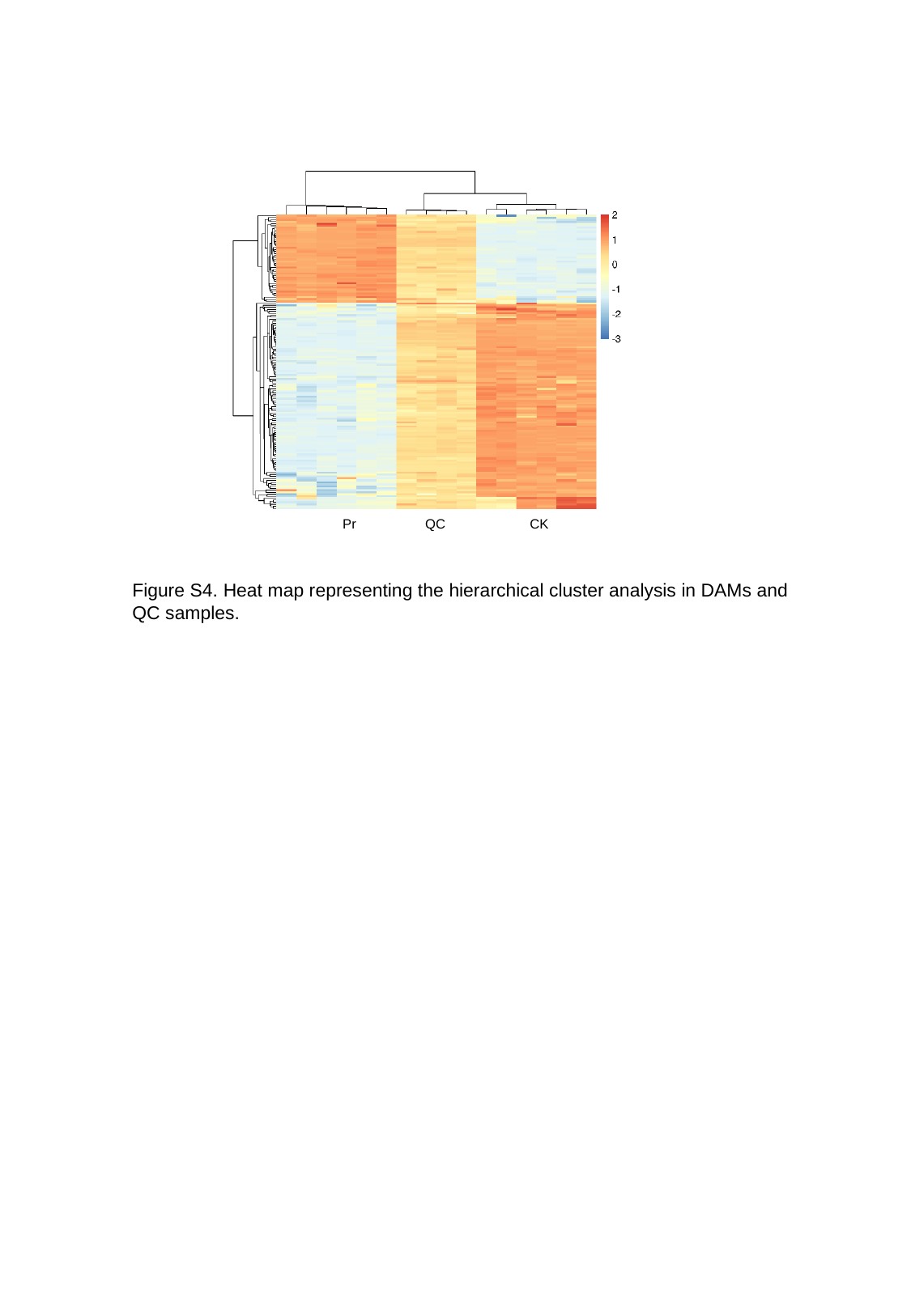

Pr QC CK
Figure S4. Heat map representing the hierarchical cluster analysis in DAMs and QC samples.

## Slide 6
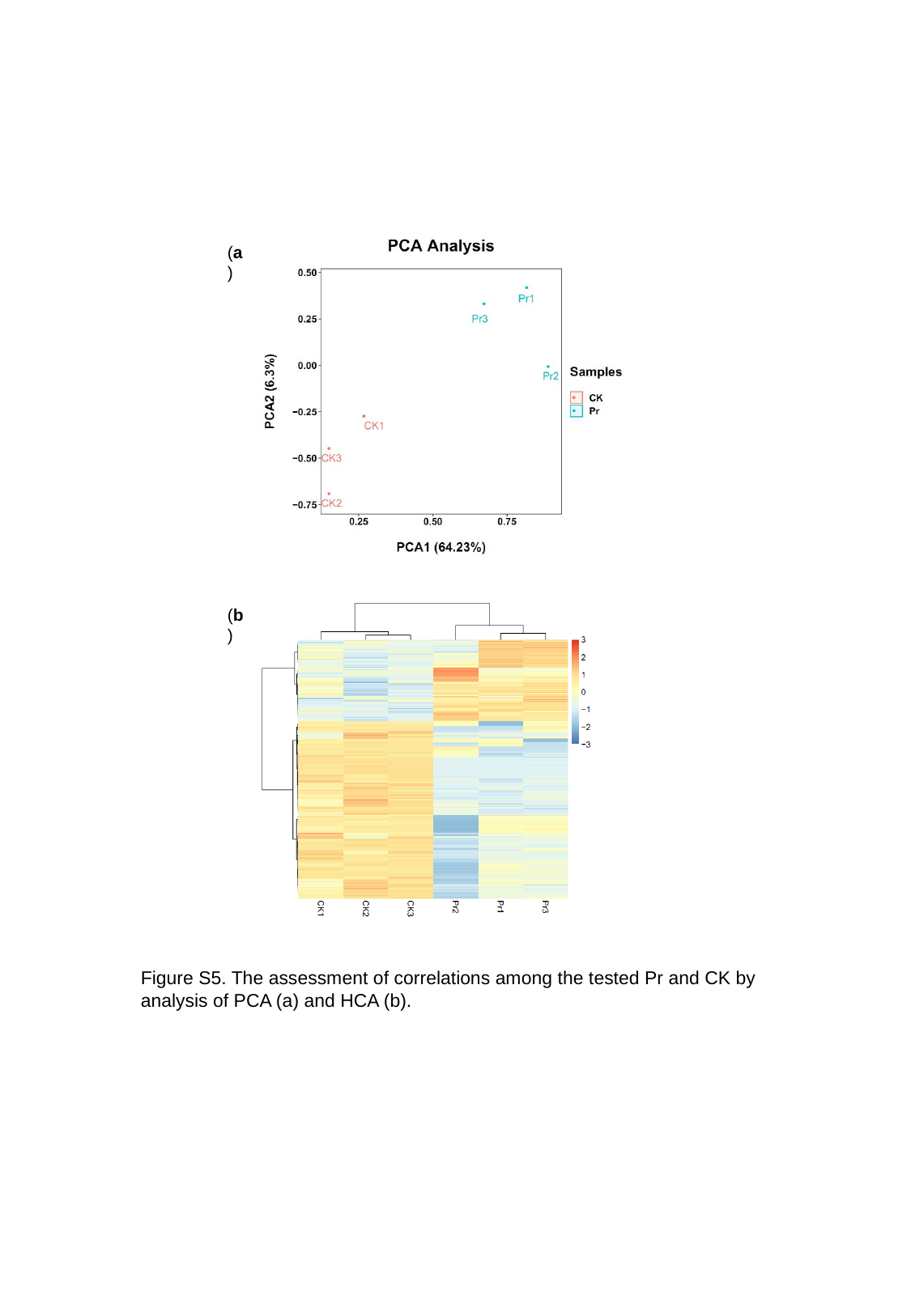

(a)
(b)
Figure S5. The assessment of correlations among the tested Pr and CK by analysis of PCA (a) and HCA (b).

## Slide 7
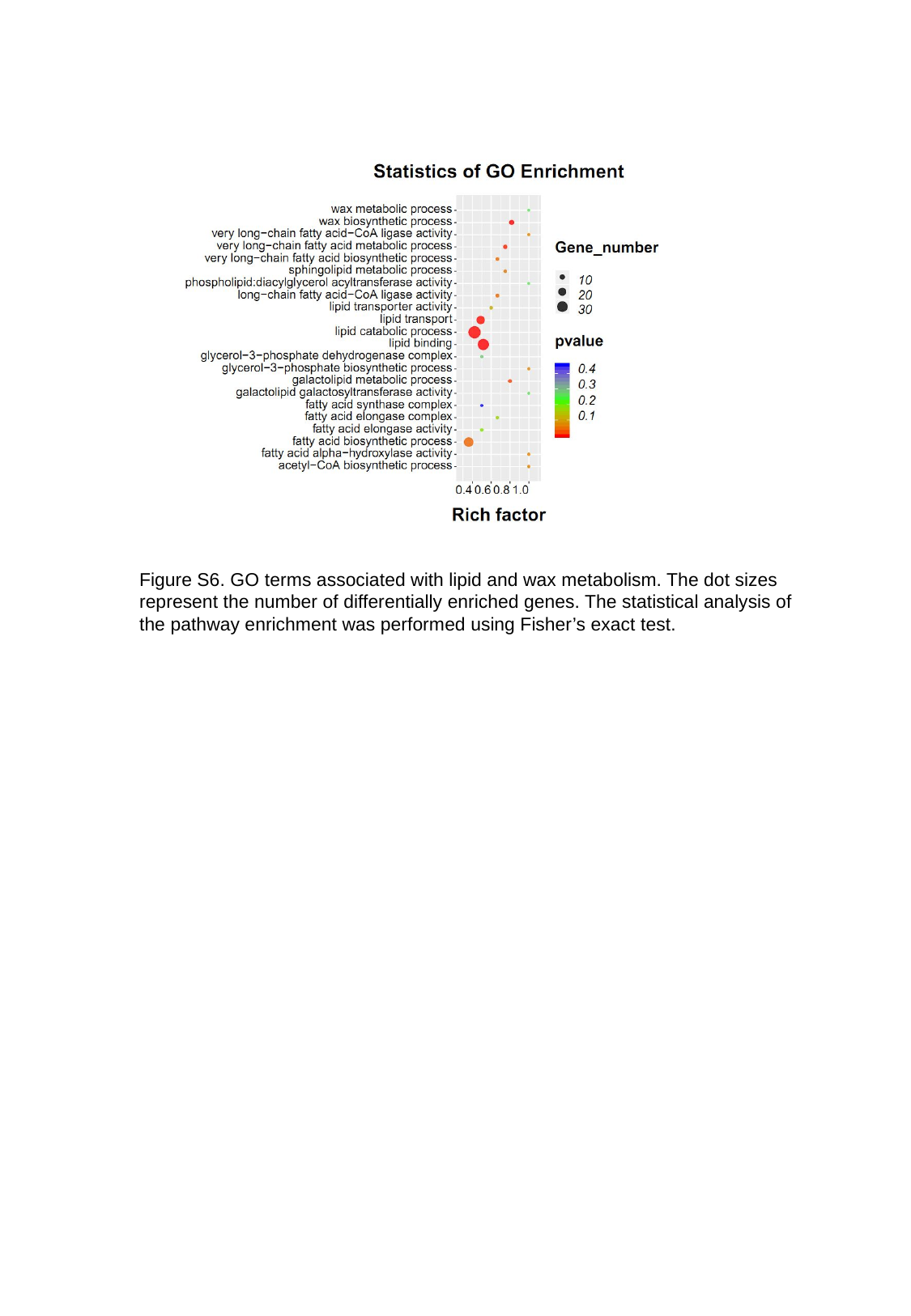

Figure S6. GO terms associated with lipid and wax metabolism. The dot sizes represent the number of differentially enriched genes. The statistical analysis of the pathway enrichment was performed using Fisher’s exact test.

## Slide 8
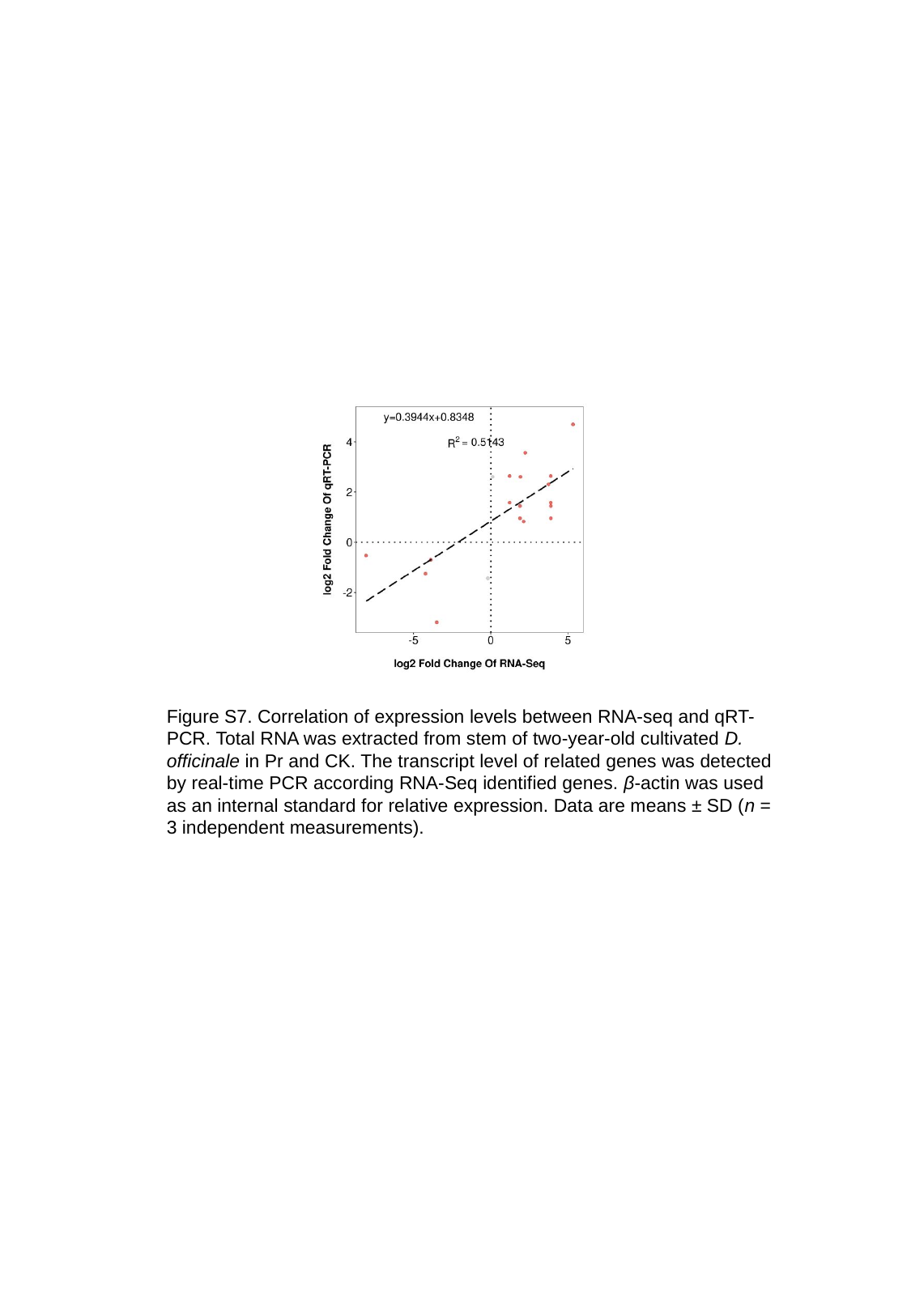

Figure S7. Correlation of expression levels between RNA-seq and qRT-PCR. Total RNA was extracted from stem of two-year-old cultivated D. officinale in Pr and CK. The transcript level of related genes was detected by real‐time PCR according RNA-Seq identified genes. β‐actin was used as an internal standard for relative expression. Data are means ± SD (n = 3 independent measurements).

## Slide 9
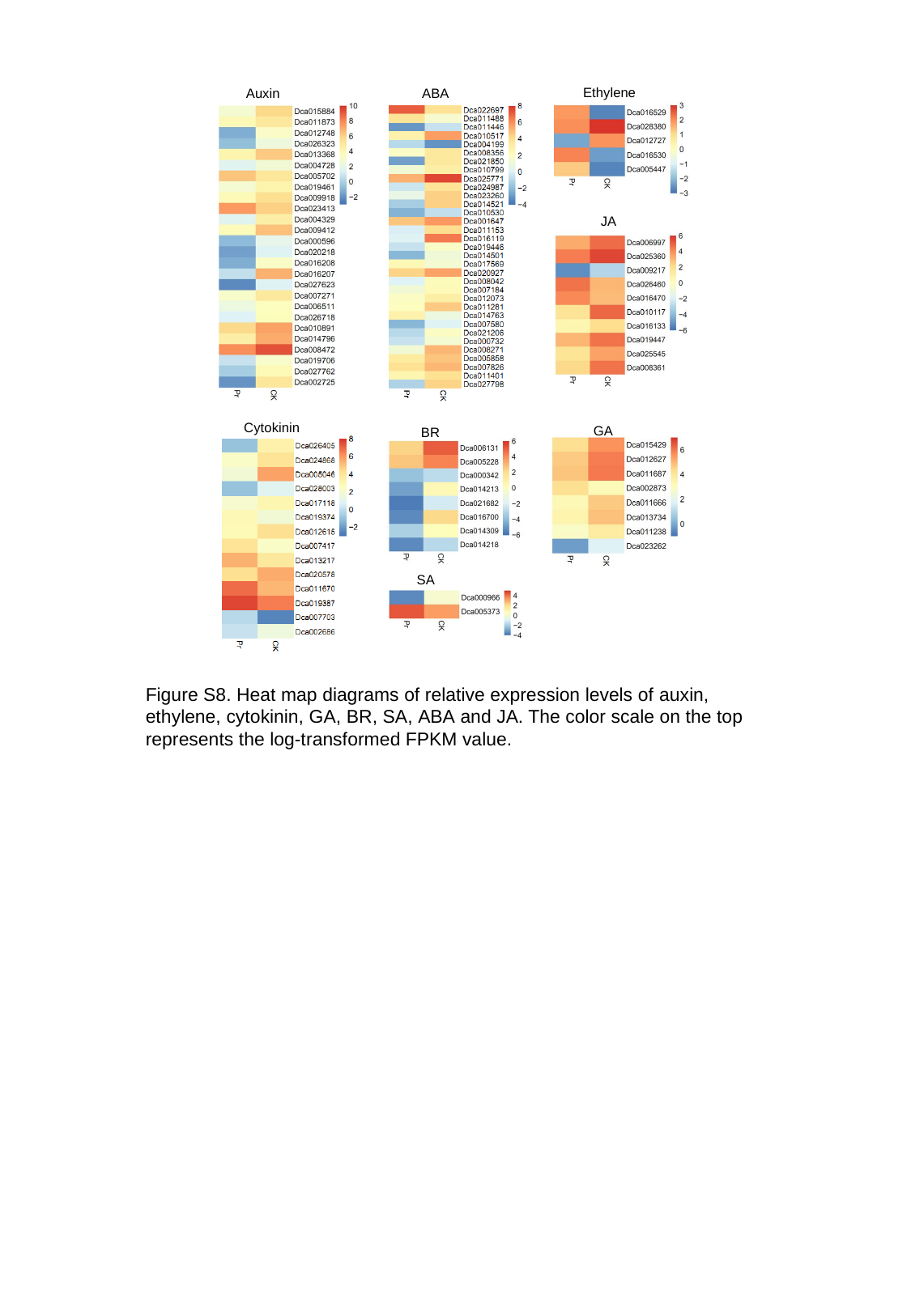

Ethylene
ABA
Auxin
JA
Cytokinin
GA
BR
SA
Figure S8. Heat map diagrams of relative expression levels of auxin, ethylene, cytokinin, GA, BR, SA, ABA and JA. The color scale on the top represents the log-transformed FPKM value.
